# Protein-DNA interactions define the mechanistic aspects of circle formation and insertion reactions in IS*2 *transposition

**DOI:** 10.1186/1759-8753-3-1

**Published:** 2012-01-26

**Authors:** Leslie A Lewis, Mekbib Astatke, Peter T Umekubo, Shaheen Alvi, Robert Saby, Jehan Afrose, Pedro H Oliveira, Gabriel A Monteiro, Duarte MF Prazeres

**Affiliations:** 1 Department of Biology, York College of the City University of New York, Jamaica, New York 11451, USA; 2 Program in Cellular, Molecular and Developmental Biology, Graduate Center, City University of New York, New York, New York 11016, USA; 3 Johns Hopkins University, Applied Physics Laboratory, Laurel, Maryland, 20723, USA; 4 Accera Inc., Broomfield, Colorado 80021, USA; 5 Ross Medical School, Roseau, Dominica; 6 Department of Occupational Therapy, York College of the City University of New York, Jamaica, New York 11451, USA; 7 Skirball Institute of Biomolecular Medicine, New York University School of Medicine, New York, New York, 10016, USA; 8 Department of Bioengineering, Instituto Superior Técnico, Avenida Rovisco Pais, 1049 001, Lisboa, Portugal; 9 IBB-Institute for Biotechnology and Bioengineering, Centre for Biological and Chemical Engineering, IST, Lisboa, Portugal

**Keywords:** Curvature propensity plot data, extensive sequence-specific binding, figure-of-eight transposition intermediate, hydroxyl radical footprinting, minicircle junction, selective binding, synaptic complex, transpososome

## Abstract

**Background:**

Transposition in IS*3*, IS*30*, IS*21 *and IS*256 *insertion sequence (IS) families utilizes an unconventional two-step pathway. A figure-of-eight intermediate in Step I, from asymmetric single-strand cleavage and joining reactions, is converted into a double-stranded minicircle whose junction (the abutted left and right ends) is the substrate for symmetrical transesterification attacks on target DNA in Step II, suggesting intrinsically different synaptic complexes (SC) for each step. Transposases of these ISs bind poorly to cognate DNA and comparative biophysical analyses of SC I and SC II have proven elusive. We have prepared a native, soluble, active, GFP-tagged fusion derivative of the IS*2 *transposase that creates fully formed complexes with single-end and minicircle junction (MCJ) substrates and used these successfully in hydroxyl radical footprinting experiments.

**Results:**

In IS2, Step I reactions are physically and chemically asymmetric; the left imperfect, inverted repeat (IRL), the exclusive recipient end, lacks donor function. In SC I, different protection patterns of the cleavage domains (CDs) of the right imperfect inverted repeat (IRR; extensive *in cis*) and IRL (selective *in trans*) at the single active cognate IRR catalytic center (CC) are related to their donor and recipient functions. In SC II, extensive binding of the IRL CD *in trans *and of the abutted IRR CD *in cis *at this CC represents the first phase of the complex. An MCJ substrate precleaved at the 3' end of IRR revealed a temporary transition state with the IRL CD disengaged from the protein. We propose that in SC II, sequential 3' cleavages at the bound abutted CDs trigger a conformational change, allowing the IRL CD to complex to its cognate CC, producing the second phase. Corroborating data from enhanced residues and curvature propensity plots suggest that CD to CD interactions in SC I and SC II require IRL to assume a bent structure, to facilitate binding *in trans*.

**Conclusions:**

Different transpososomes are assembled in each step of the IS*2 *transposition pathway. Recipient versus donor end functions of the IRL CD in SC I and SC II and the conformational change in SC II that produces the phase needed for symmetrical IRL and IRR donor attacks on target DNA highlight the differences.

## Background

**Figure 1:**
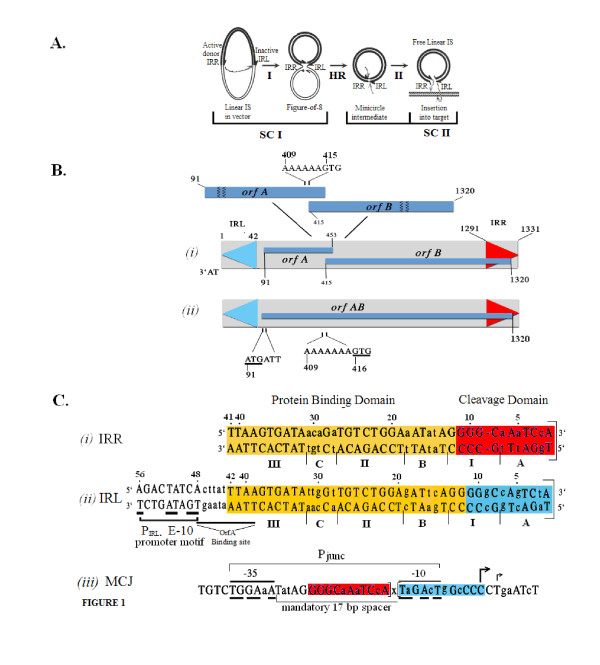
**Organization of the IS*2 *insertion sequence and its transposition pathway (modified from **[31]). **(A) **The two-step transposition pathway of IS*2*. Step I (I) occurs within SC I. Asymmetric single-strand cleavage of the IRR donor is followed by transfer to the donor-inactive IRL recipient end, creating the F-8. Host replication mechanisms convert F-8 into a covalently closed double-stranded circular intermediate, the minicircle. In Step II (II) a second synaptic complex (SC II) is assembled. Cleavage at the abutted CDs results in two exposed 3'OH groups which carry out transesterification attacks on the target DNA. **(B) **IS*2 *with IRL (blue) and IRL (red) and two overlapping open reading frames, *orfA *and *orfB*, expanded to show detail of the A_6_G slippery codons. (*i*) Translational frameshifting regulates low levels of OrfAB formation; *(ii) *high levels of the transposase are produced by altering the window to A_7_G. **(C) **Aligned sequences of *(i) *IRR and *(ii) *IRL and *(iii) *the abutted ends of theMCJ. Square brackets identify the termini of IRR and IRL. *(i) *and *(ii)*: conserved residues (within all elements) are in uppercase; diverged residues (within non-conserved elements A, B, C and I) are in lower case. The extended-10 promoter, P_IRL_, (bold underlines identify bases which match the consensus sequence) drives the events of Step I of the transposition pathway shown in panel A. Residues 39 to 48 are shown in these studies to include the binding sequence for the repressor function of Orf A [20]. (*iii*): abutted ends at the MCJ form a more powerful promoter (P_junc_) which indispensably controls the events in Step II. The only functional form of P_junc _contains a single base pair spacer (x) which creates its mandatory 17 base pair spacer. CD: cleavage domain; F-8: figure-of-eight; IRR/IRL: right and left inverted repeats; IS: insertion sequence; SC: synaptic complex; MCJ: minicircle junction.

IS*2*, a 1.3 kb transposable element, is a member of the large and widespread IS*3 *family of insertion sequences (IS) ([1,2] see also ISfinder: http://www-is.biotoul.fr/is.html). Transposition mechanisms in the IS*3 *family can be described as a two-step copy-and-paste process [3], in contrast to both classical cut-and-paste and replicative paradigms [4-6]. Although transposases of two IS*3 *family members, IS*911 *[7-9] and IS*2 *[10,11] were originally shown to facilitate transposition by catalyzing the two distinct reactions whose steps are shown in Figure 1A, there is strong evidence for the existence of this pathway in other IS*3 *family members such as IS*3 *[12,13] and IS*150 *[14] as well as for its more widespread use in the IS*30 *[15,16], IS*21 *[17] and IS*256 *[18] families of insertion sequences. In general in these families, Step I involves a cleavage and joining reaction between the ends, one of which (the optional donor) is cleaved and participates in an asymmetric, intrastrand, strand-transfer reaction to a phosphodiester bond in host DNA near the other end (the recipient). The product is a branched structure, the figure-of-eight (F-8) transposition intermediate [7,11,16] in which two abutted single-stranded ends are separated by an interstitial spacer of one or more bases. The F-8 is then converted by host cell replication mechanisms [3] to a covalently closed double-stranded transposition intermediate, the minicircle, (Figure 1A) whose abutted ends, separated by the spacer, comprise a reactive junction, the minicircle junction (MCJ). Minicircle insertion into the target occurs in Step II (Figure 1A) and requires that both ends function as donors [10,19]. Here, the reactive junction is the substrate for strand transfer reactions: it is cleaved at the abutted termini of the ends, creating 3'OH groups which undergo symmetrical transesterification attacks on target DNA. This results in the insertion of the element flanked by its direct repeats; see Rousseau *et al*., [2] for a detailed review.

The ends of IS*2 *are 41 bp and 42 bp right and left imperfect inverted repeats (IRR and IRL; Figure 1B) respectively; between these ends the IS encodes two overlapping reading frames, O*rfA *and O*rfB *(Figure 1B, i). OrfA is a 14 kDa protein which has been reported in IS*2 *[20] to bind to a sequence just upstream of the weak indigenous extended-10 promoter (P_IRL_-[10]) located just inside the left end (IRL) of the element (Figure 1C, ii). This weak promoter regulates the expression of IS*2 *proteins in Step I. The function of OrfB is unknown but a fusion protein OrfAB, the functional transposase (TPase), is generated by programmed -1 translational frameshifting [13,21,22] at a sequence of slippery codons (the A_6_G frameshift window in IS*2*), located near the 3' end of *orfA *(Figure 1B, i). Mutation of this window to A_7_G in IS*2 *(Figure 1B, ii) produces OrfAB as the predominant species [11,23]. When the IS*2 *ends are aligned (Figure 1C, i, ii), they show four non-conserved elements (I, A, B and C) and two conserved elements (II and III) which play critical roles in the transposition mechanism. Elements A and I comprise a cleavage domain (CD) and B, II, C and III, a protein binding domain (PBD). The differences in the sequences of the two ends are related to their donor and recipient end functions (see below) in Step I [24].

Several features distinguish circle formation and its consequences in IS*2 *from those in other IS*3 *family members. The reaction is physically as well as chemically asymmetric in that the right end functions uniquely as the donor or transferred end and the left end serves exclusively as the functional recipient end. This asymmetry is not unique to IS*2*, having also been demonstrated in copies of IS*256 *in Tn*4001 *[18]. Recipient end function in IS*2 *is partially defined by the accuracy with which the joining reaction occurs. Abutted ends at the MCJ (Figure 1C, iii) are separated by a one or two base pair spacer with a ratio of 90% to 10% [11,24] but functional minicircles are limited to those with a single base pair spacer. This is so because creation of the MCJ in IS*2 *assembles a promoter, P_junc_, [25] which has an absolute requirement for a 17-nucleotide promoter spacer (Figure 1C, i, ii) that is conferred by the one base pair MCJ spacer. This more powerful P_junc _is essential for and drives transposase reactions in Step II [10]. MCJ promoters with spacers of two or more base pairs are completely non-functional.

We concluded from earlier studies that differences in length and sequence of the two ends of IS*2 *in Step I are responsible for the restriction of donor and recipient end functions to IRR and IRL respectively [24]. Differences in length are related to the correct positioning of the shorter donor end (IRR) in the catalytic pocket. However, random mutation in the A element of the A^IRR ^sequence in an IRR CD eliminated minicircle production, while similar changes in A^IRL ^in an IRL CD had no effect on the efficiency of minicircle formation; this result implied that extensive sequence-specific protein affinity for the A element was important in defining donor function but not recipient end function. For the B element, mutations in the B^IRR ^sequence also eliminated minicircle formation, implicating sequence-specific protein affinity. Additional domain swapping experiments involved the substitution of a 6 bp B^IRL ^sequence in an IRR derivative, which did not change the length of IRR (Figure 1C, i and 1C, ii). This reduced but did not eliminate IRR donor activity, implying that the protein had a weaker affinity for B^IRL^. Further evidence for some protein interaction with the B^IRL ^sequence is that in IRL, its mutation (a triplet of point mutations) all but eliminated minicircle formation. These results suggested that the degree of sequence-specific interaction of the protein for sequences in or near the CDs may also be related to donor and recipient end functions; in an IRR end, extensive interaction of the protein with A^IRR ^and B^IRR ^would be required for the donor function; however, in IRL the lack of extensive interaction of the protein with A^IRL ^and a weak affinity for B^IRL ^may contribute to recipient end identity.

Additional data from experiments with A^IRL ^threw light on this supposition. First, in an IS*2 *mutant with two IRR ends, the increase in length of one IRR by a single base pair alone was necessary and sufficient to convert it to a recipient end with no donor function. However, the addition of A^IRL ^was absolutely essential for the accuracy of recipient end function. Furthermore, alteration of any one of three non-conserved nucleotides in positions 2, 5 and 7 in A^IRL ^(Figure 1C, ii) that made the sequence more like that of the IRR CD reduced the accuracy of the joining reaction in Step I by increasing MCJ spacer size. We posited then, that the non-conserved base pairs in A^IRL^, through some interaction with the protein, were responsible for the accuracy of recipient end function by correctly positioning the IRL CD *in trans *in the vicinity of the IRR CD to generate a single interstitial base pair between the abutted ends. (See the Results and discussion section for a complete analysis of all factors which define recipient end function.) It is interesting that mutation of position 2 of IRL, which converted the TA3' terminal dinucleotide to the CA3' consensus in the IS*3 *family, did not confer functional donor activity on IRL [24], due, among other factors, to its incorrect positioning in the cognate catalytic center (CC). Finally, although the features described above for IRL define its accuracy as a recipient end, the sequence of the flanking host DNA can also play a role in determining spacer size [24], implying that the host DNA sequence adjacent to IRL is also involved in some kind of interaction with the TPase.

**Figure 2:**
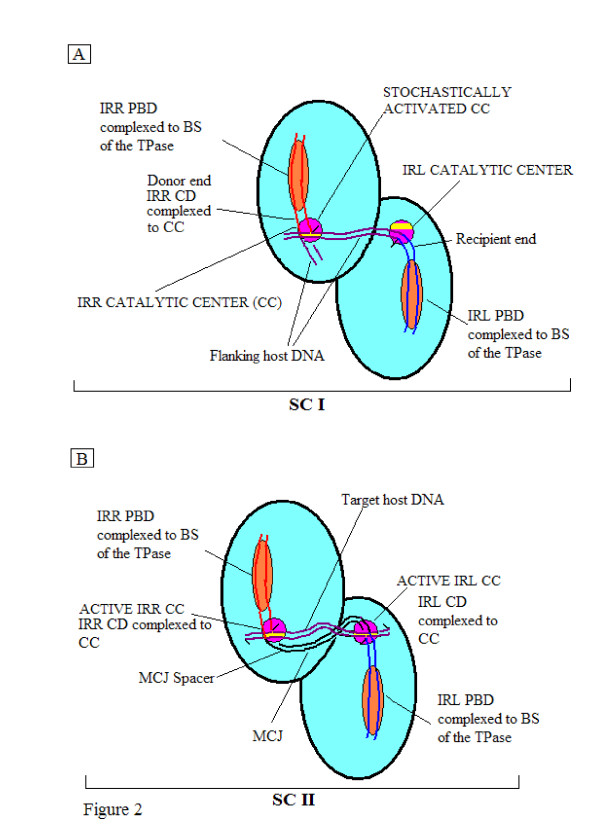
**Idealized schematic representations of synaptic complexes (SC I and SC II) of circle-forming insertion sequences**. Each complex is shown as a dimer (aqua ovals) with a BS (orange) and a CC (purple). Each IR is complexed with its PBD (red for IRR and blue for IRL) to the BS of its monomer, and its CD bound *in cis *to the CC. **(A) **In SC I, at one stochastically activated CC (IRR in this case) the CD is cleaved at its 3' end, exposing a 3'OH group (black half arrow) which, in a transesterification reaction, attacks the host DNA (maroon; flanking the other (IRL) end), which is bound non-specifically to the CC in a tract (yellow band) designated for target or host DNA. The reaction creates the branched figure-of-eight structure (precursor of the minicircle) with an interstitial sequence of host DNA (which will become the MCJ spacer between the abutted ends) equal in length to the distance between the two CCs. **(B) **In SC II, the two ends are complexed as in SC I with the MCJ spacer (black) spanning the distance between two active CCs. At each activated CC the 3' end of each IR is cleaved and the exposed 3'OH groups (broken strands with black half arrows) carry out concerted transesterification attacks (yellow dots) on target DNA (maroon) which is complexed through non-specific binding to the CCs (yellow tracts). This initiates the insertion event and the resulting direct repeats which are signatures of insertion will be equal in length to the MCJ spacer. BS: binding site; CC: catalytic center; CD: cleavage domain; IRR/IRL: right and left imperfect, inverted repeats; MCJ: minicircle junction; PBD: protein binding domain; SC: synaptic complex.

Mechanistically, in elements with F-8 transposition intermediates, the right and left ends of the linear element (attached to flanking host sequences) are organized with the transposase in Step I into a nucleoprotein complex known as the transpososome or Synaptic Complex (SC) I [26,27]. We have proposed [24] that generally for IS*3 *family members, each monomer of this complex, viewed as a dimer, would possess a binding site (BS) occupied by the PBD of one end and a cognate CC at which the CD would be bound *in cis*, that is, with PBD and CD bound on the same monomer (Figure 2A). By a stochastic process either one of these CCs would be activated to generate the donor end. This optional donor is cleaved and the exposed 3'OH group attacks a phosphodiester bond in the host DNA adjacent to the CD of the opposite end at a position corresponding to the distance between the two CCs. This forms the interstitial or MCJ spacer (equivalent to the size of the direct repeat) between the abutted single-stranded ends. In SC II transpososomes, the CDs of the MCJ separated by the short spacer would be bound *in cis *at the two active CCs (Figure 2B). There, sequential or concerted cleavages would generate 3'OH groups, whose symmetrical attacks on the target DNA appropriately positioned at the CCs would effect insertion and the formation of direct repeats. This general scenario would explain the similarity between the sizes of the MCJ spacer and the direct repeats.

For IS*2*, however, we proposed that in SC I, given the donor-inactive IRL, the 5 bp distance between the two CCs and the one base pair MCJ spacer size, the IRL CD would have to be positioned near the single active CC at which the IRR CD was bound to facilitate the joining reaction. For SC II, we proposed that the abutted CDs separated by a single base pair would also be complexed at a single active cognate IRR CC (the first transition state) and that a series of cleavage-triggered conformational changes would result in each CD *cis*-bound at its cognate CC (as shown in Figure 2B). It is important to note, however, that other factors may play an important part in this process in SC II, such as the role of the IS*911 *OrfA, which has been shown in *in vitro *assays to stimulate insertion principally into DNA targets devoid of IS*911 *end sequences [28]. Nevertheless, in these ISs, the assembly of intrinsically different SC I and SC II transpososomes appears to be necessary [24,26]. This conclusion is applicable to circle forming elements in the IS*3 *family which use the two-step pathway, for example, IS*3 *[12,13] IS*150 *[14] and IS*911 *[7,8], where MCJ spacer size is similar (2 bp to 4 bp) to that of the direct repeat (see Figure 2). It is particularly true for the SC II in IS*2 *[24] and in IS*256 *in Tn*4001 *[18], where physically asymmetric Step I reactions have been described and where the acquisition of donor function by the recipient end, lacking in Step I, is essential. Similar thinking would apply to IS*21 *[29] and IS*1665 *[30] where, as is the case in IS*256*, the interstitial MCJ distance is less than the size of the direct repeat. In this study we have tested these hypotheses with hydroxyl radical footprinting analyses of Step I complexes of IS*2 *and by comparative footprinting analyses of covalently joined and pre-cleaved (or nicked) MCJ substrates in SC II.

**Figure 3:**
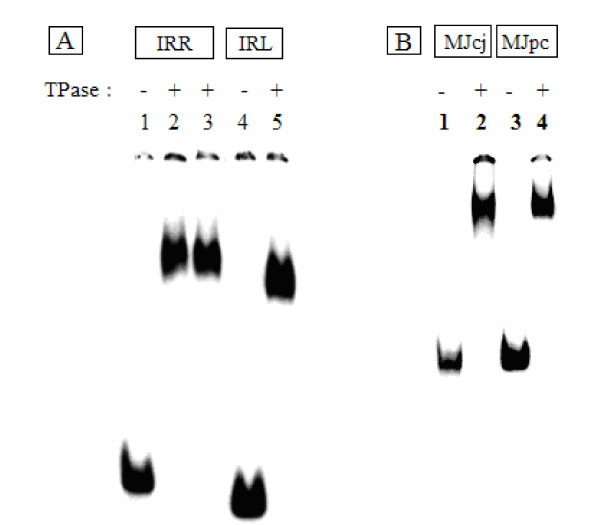
**Protein-DNA complexes visualized by gel retardation assays run on 5% polyacrylamide gels**. For each lane, 80 nM of partially purified IS*2*OrfAB-GFP was reacted with 2 nM ^32^P-labeled annealed oligonucleotides containing cognate DNA sequences from IRR, IRL, or the minicircle junction substrates MJcj and MJpc. The reactions were incubated at room temperature (20°C) for 30 min, loaded onto the gel at 4°C and run at 120 mA. **(A) **Lanes 1 to 3: 87-mer IRR; 4 to 5: 79-mer IRL. Different preparations of the protein were used in lanes 2 and 3. The gel was run for 1400 Vhr. **(B) **Lanes 1 and 2: 114-mer MJcj; 3 and 4: MJpc. The gel was run for 920 Vhr. IRR/IRL: right and left inverted repeats; MJcj: covalently joined minicircle junction substrate; MJpc: precleaved minicircle junction substrate.

The 46 kDa IS*2 *transposase is expressed in active soluble form with great difficulty and solubilized, renatured, highly purified preparations bind poorly to oligonucleotides containing cognate IRR and IRL sequences. A TPase derivative, C-terminal-tagged with GFP, produced a full length soluble 74 kDa OrfAB-GFP fusion protein under native conditions. When purified to near homogeneity, this fusion protein also bound poorly to similar oligonucleotides even though it is fully active *in vivo *[31]. These results of poor or low binding efficiency of the full length transposase are similar to those for IS*911 *[26,27,32], IS*30 *[33,34] and IS*256 *[35]. As a consequence, a comparative biophysical analysis of protein-DNA interactions in fully formed Step I and Step II complexes with protein bound to both binding and cleavage domains of the ends has not been reported for this group of circle-forming insertion sequences. However, soluble, active preparations of partially purified IS*2 *OrfAB-GFP produced complexes in which both the DNA BD or BS and the CC of the protein bound very efficiently to cognate IRR sequences in linear oligonucleotides [31]. We have now successfully used complexes created with single-end and MCJ substrates (Figure 3) to generate hydroxyl radical footprinting data.

We show here that the footprinting patterns of both IRR and IRL single ends of IS*2 *reveal bipartite structures. They differ in that the IRR CD is strongly and extensively protected while the IRL CD is only selectively or intermittently bound by the protein. We propose a model in which non-specific and/or selective binding to the adjacent host sequence and selective binding to the IRL CD act additively in SC I to promote binding of the IRL CD *in trans *at the active cognate IRR CC. In SC II, extensive protection of both the IRL and the abutted IRR CDs, separated by a single base pair, suggests binding at a single active cognate IRR CC with the IRL CD bound *in trans*, creating the first phase of the SC. Our data suggest that sequential cleavages (associated with small conformational changes) at the 3' termini of IRR and IRL at this active CC trigger a conformational change that leads to transition to a second phase; that is, each CD complexed *in cis *to its own active cognate CC. In addition, the location of enhanced residues indicative of distortion or bending of DNA, corroborated by curvature propensity plot data, have helped gain insight into the paths of the IRL DNA which facilitate binding *in trans *within the architecture of SC I and SC II transpososomes.

## Results and discussion

### Footprinting the single ends of IS*2*

Hydroxyl radical footprinting was carried out using 87 bp (R87) and 79 bp (L79) radio-labeled dsDNA substrates containing the 41 bp sequence of IRR and the 42 bp sequence of IRL, respectively. The substrates were prepared as annealed oligonucleotides with the labeled strand as the footprinting target (see the Methods section). The transposase was overexpressed from pLL2522, the plasmid with the *orfAB::GFP *fusion construct, and partially purified by nickel-nitrilotriacetic acid (Ni-NTA) affinity chromatography [31]. Mutational studies with this partially purified protein (specifically null mutants with a complete loss of binding proficiency), indicated strongly that the observed binding reactions did not result from trace amounts of the IS*2 *Tpase from chromosomal copies of the element. In addition, two sets of results suggest that the presence of the GFP tag affected neither the binding properties nor the activity of OrfAB. First, *in vivo *transposition frequencies of the tagged protein are statistically identical to those of the native protein [31]; secondly, in a cleavage assay [36], complexes formed in-gel with a mixture of 87-mer IRR and 50-mer IRR substrates, generated the 95 nucleotide and 114 nucleotide high molecular weight recombination products predicted for paired-ends complexes formed by a chemically active protein activated with Mg^2+ ^(Additional file 1). This latter result and footprinting data from complexes formed with the MCJ substrates in which both ends are protected along their lengths, indicate that fully formed complexes are generated by the OrfAB-GFP protein and that paired-ends complexes composed of at least dimers are being formed. For footprinting reactions, the protein-DNA complexes, initially visualized in the gel retardation assays shown in Figure 3A, were formed in solution and subjected to cleavage reactions at room temperature (20°C) prior to fractionation on 8% polyacrylamide sequencing gels.

**Figure 4:**
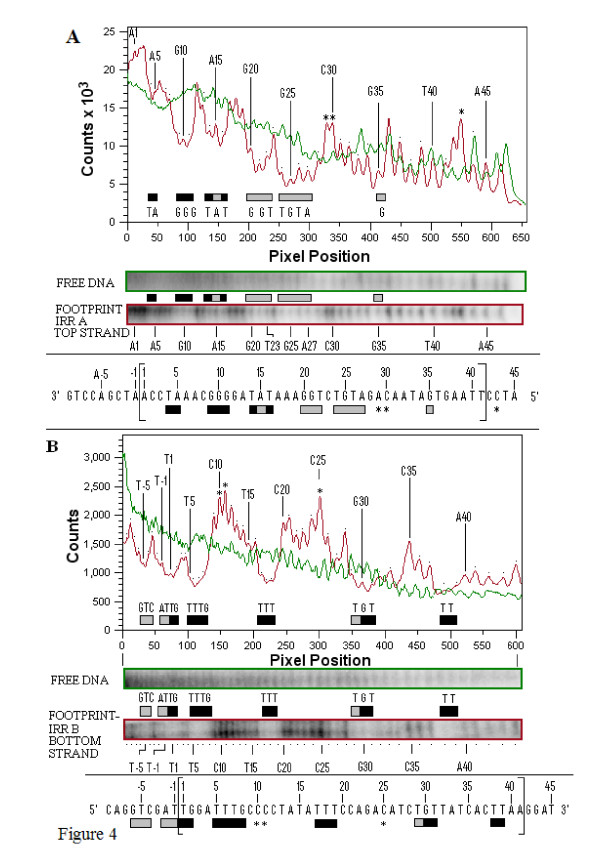
**Hydroxyl radical footprinting of the top (IRRA) and bottom (IRRB) strands of the IS*2 *IRR**. **(A) **Quantitative analysis panel, with tracings (derived from the color-coded gels immediately below the panel) showing relative intensities of bands from the footprinted, cleaved, bound top strand of IRR, (IRRA-red tracing) and the control, cleaved, unbound, free DNA, (green tracing). The protection profile is shown as horizontal bars within the panel identifying troughs of weakly (grey) and strongly (black) protected residues that are significantly below the green control. Determination of strong and weak protection was based on the combined analysis of visual evidence of a band and the absence or presence of peaks within the troughs. Visual absence of a band coupled with absence, or only a suggestion, of a peak defined strong protection. A faint band which showed a small peak within a trough defined weak protection. Bands and peaks are numbered (1 to 41) from the outer (3') end of IRRA to the inner end. Individual peaks are identified by dots and numbered vertical lines identify the nature of every fifth base. Asterisks identify enhanced residues whose red peaks rise significantly above those of the green control. The sequence of IRRA, shown below the panel was used to annotate the peaks in the upper panel and the bands in the color coded lanes. Nucleotides are numbered as described above. The IRR sequence within the large brackets, is flanked by host DNA at the outer (3') end of the terminus (-1 to -9) and the sequence of IS*2 *adjacent to the inner end of the terminus (42 to 45). **(B) **Quantitative analysis panel showing relative intensities of bands from the footprinted IRRB DNA (red) and the control DNA (green) derived from the gels shown immediately below the panel as described in part (a). IRR: right inverted repeat.

**Figure 5:**
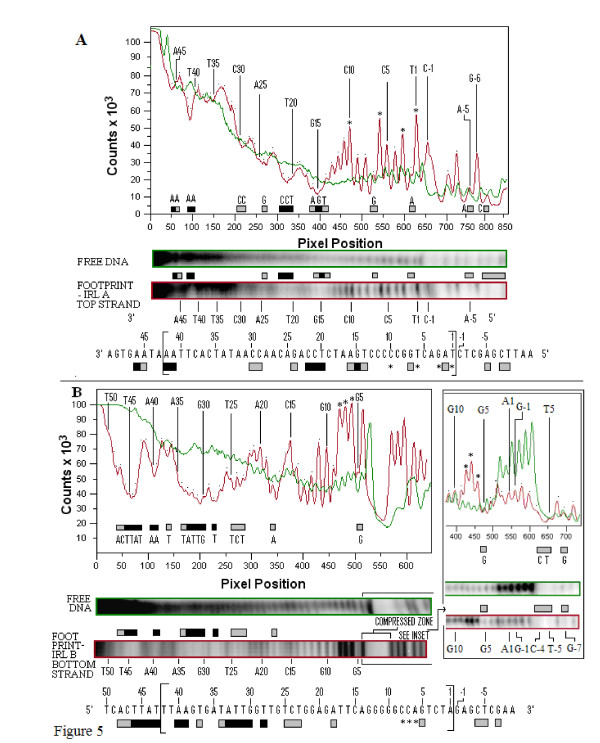
**Hydroxyl radical footprinting of the top (IRLA) and bottom (IRLB) strands of the IS*2 *IRL**. **(A) **Quantitative analysis panel showing relative intensities of bands from the footprint of IRLA (red) and the control DNA (green) as described in Figure 4. Determination of the protection profiles is as described in Figure 4. Bands and peaks are numbered (1 to 42) from the outer end of the terminus of IRR (the 5' end of the strand) to the inner end. The sequence of the top strand of IRL is shown below the panel. The IRL sequence (within large brackets and numbered as described above) is flanked by host DNA at the outer end of the terminus (-1 to -11) and the sequence of IS*2 *adjacent to the inner end of the terminus (43 to 50). **(B) **Quantitative analysis panel showing relative intensities of bands from the footprint of IRLB (red) and the control DNA (green). The zone of compression which masks the footprinting pattern from G5 to A-9 is shown more clearly in the inset. IRL: left inverted repeat.

**Figure 6:**
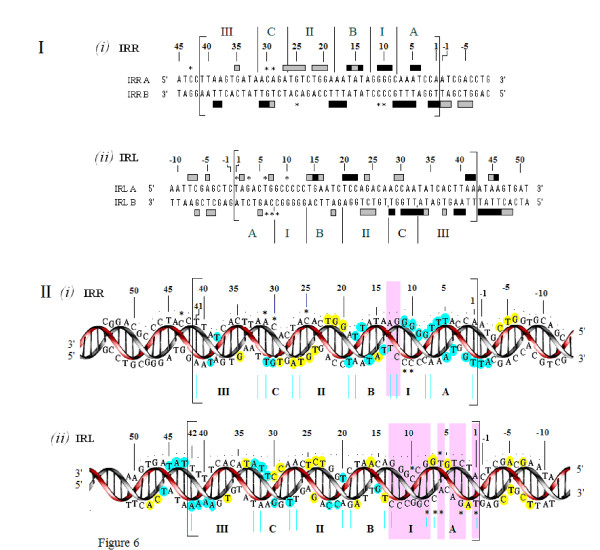
**Summary of footprinting patterns of the double-stranded right and left ends of IS*2***. **(I) **Double-stranded sequences of IRR and IRL are shown within the large brackets, numbered from the outside ends to inside ends as described in Figure 4. Protected nucleotides, strong (black) and weak (grey), (as described in Figure 4) are indicated by filled horizontal bars. Enhanced nucleotides are indicated by asterisks. Conserved and non-conserved elements are as described in Figure 1. **(II) **Three-dimensional representations of the protection patterns shown in part I. For IRR, the red helix represents the lower strand (IRRB- 5'TGGATT... TTAA3') and the gray helix, the upper strand (IRRA- 5'TTAA... AATCCA3'). For IRL the red helix represents the upper strand (IRLA - 5'TAG... TTAA3') and the grey helix the lower strand (IRLB- 5'TTAA... CTA3'). Strong and weak protections are shown as filled blue and yellow circles, respectively. Vertical purple shaded bars highlight the difference between the selective binding of the cleavage domain of IRL, illustrated by intermittent binding of three of the eleven nucleotides and the extensive protection of the cleavage domain of IRR with a single gap at its inner end (see text). Annotation is as described in part I. In both parts, numbering is as described in Figure 4. The inside terminus of IRL shows protection of the sequence numbered 39 to 48 that includes the proposed binding sequence for the repressor function of the OrfA protein [20]. The 5'TGAT3' sequence of base pairs 48 to 51 represents the first four bases of the weak indigenous extended-10 promoter (P_IRL_, see Figure 1) located adjacent to the inner end of IRL. IRR/IRL: right and left inverted repeats.

Sequencing gel data of each of the strands of IRR and IRL, composed of three side-by-side lanes showing the guanine and adenine (G+A) Maxam-Gilbert sequencing reactions, the cleaved unbound (free) DNA and the cleaved bound (footprinted) DNA, are shown in Additional file 2. Comparative densitometer tracings from sequencing gels of the footprinted and free DNA lanes for the top and bottom strands of the IRR substrate are shown in Figure 4A, B. Similar results for the IRL substrate are shown in Figure 5A, B. The most consistent protection patterns, based on the gel data and the densitometer tracings, are summarized below the panels. The protection patterns for the double-stranded molecules are summarized in Figure 6I, II. Numbering of the bases in all figures starts at the outside ends of IRR and IRL and proceeds to the inside ends. The amount of DNA in the bands in the footprinted reactions in all of these experiments is a reflection of the extreme efficiency of the binding of the DNA by the protein (Figure 3).

Data summarized in Figure 6 indicate that an 11 bp sequence at the outside end of IRR that makes up the cleavage domain (the A and I elements) is strongly protected by the transposase. Strong protection is also observed for the B element at the outside end of the PBD of IRR, although a gap at base pairs 12 and 13 separates the IRR cleavage domain from the B^IRR ^(Figure 6II, i). Extensive but weaker interactions are associated with elements at the inside terminus of the end. On the other hand, the first 11 bp of the IRL CD (elements A and I) are only intermittently contacted by the protein (Figure 5), at positions 2, 5 and 7, the same residues shown from earlier mutation studies to affect the accuracy of the joining reaction. In addition, in B^IRL^, the residues are more weakly bound than those of B^IRR ^(summarized schematically in Figure 6II, ii). Thus, the cleavage domain of IRL is not extensively protected by the transposase in Step I. We refer to the intermittent binding of the IRL CD as selective binding, which describes the interaction of the protein with a few residues of the sequence of the recipient end in order to ensure the accuracy of the joining reaction. These results support conclusions reached from earlier mutational studies [24] that A^IRR ^and B^IRR ^are important binding targets in IRR for the TPase, that B^IRL ^would be bound with lower affinity, that A^IRL ^would not be the subject of sequence-specific binding and that its residues 2, 5 and 7 might have a unique type of interaction with the TPase.

### The different bipartite footprinting patterns of the single IRR and IRL ends are related to their functions in the Step I transpososome

Our results provide physical confirmation of earlier genetic data that the functionally bipartite ends are composed of an outer 11 bp cleavage domain and an inner protein binding domain [24]. We conclude that the strong protection of the CD of IRR, likely protected at two major grooves (Figure 6II, i), results from sequence-specific binding by the catalytic center of the protein and propose that it creates a stable complex which enables accurate cleavage of the donor end to take place. We arrive at this conclusion by taking into account the recent results of mutations in the catalytic center of the transposase, in which alteration of three residues in the beta strands and alpha helices of the CC generated partially dissociated complexes in electrophoretic mobility shift assays (EMSAs). This suggested that a loss of affinity of this part of the protein for the DNA substrate had occurred. Similar mutant phenotypes were also observed for mutations in the binding domain of the protein, indicative of two distinct but interdependent binding capabilities of the protein [31].

We propose that in both IRR and IRL, the B elements, which are also bound extensively at major grooves, together with the II elements comprise the major targets of the BD of OrfAB (Figure 6II). This is not unlike the situation in IS*911 *[26] and IS*30 *[37]. In the former, the β domain of the ends was specifically bound and protected by a truncated N-terminal fragment of the transposase, whereas in IS*30 *the central region of the ends was protected by a similarly truncated derivative. In IRL of IS*2*, binding of B^IRL ^is weaker than that of B^IRR^, a result that is supported by data from earlier mutation studies which showed the inability of B^IRL ^to maintain normal levels of donor activity in an IRR end [24]. This weaker protection pattern may be related to the need to allow the tip of IRL (that is, the CD) to be bent (see below).

The differences in the protection patterns of IRR and IRL in SC I correspond to their functions. While the extensive protection of the B element and of the CD of IRR creates and stabilizes an enzymatically competent complex, we propose that the selective binding to the IRL CD and non-specific and/or selective binding to the adjacent host DNA (Figure 6, positions -1 to -8) act additively to direct the CD away from a *cis *interaction with its cognate CC by bending the DNA to facilitate binding *in trans *at the active CC, while simultaneously determining the accuracy of the joining reaction. An additional aspect of the data in Figure 6 appears to support this idea. The cleavage domain of IRL shows a relatively high frequency of enhanced residues (six of the eleven positions), compared to its PBD. This suggests that the IRL CD in SC I is distorted because it may need to be bent by the protein. It is interesting that in both substrates L79 (residues 1,3,6,7,8 and 10) and R87 (residues 9 and 10), the enhanced residues are associated with a series of base pairs comprising a guanine/cytosine-rich tract within the CDs (positions 7 to 13 in IRL and 8 to 12 in IRR; Figure 6), a sequence which facilitates bending of the DNA [38,39]. In support of this idea are results from an IS*2 *derivative with multiple transversion mutations at positions 8 to 12 of IRL (I^IRL^), in which minicircle formation was completely abolished [24], although current results do not show protection of these residues by the protein.

We interpret these data as suggesting that the IRL CD is positioned *in trans *and juxtaposed to the active CC occupied by the *cis*-bound IRR CD, in a tract which is probably that used for non-specific binding to the host DNA. The importance of non-specific and/or selective binding of the adjacent host DNA by the protein receives support from our earlier studies, which indicated that the nature of the host DNA flanking the recipient end can play a role in determining MCJ spacer size [24], as well as from a more recent report of the binding efficiency of a truncated version of the IS*911 *OrfAB (residues 1 to 149). This derivative bound much less efficiently to a 36 bp substrate containing only the IRR sequence than to a longer 100 bp substrate, due, they proposed, to the non-specific binding capability of the transposase [27]. This interpretation of the architecture of the IS*2 *SC I is further supported by data from studies in which mutated IS*2 *derivatives with two left ends produced no minicircles [24]. When complexes are formed *in vitro *with only DNA of the left end, several factors would then work against either IRL functioning as a donor (that is, bound *in cis *at its cognate CC): selective rather than extensive binding of their CDs; the non-specific and/or selective binding of adjacent host DNA; their longer length (one bp) than donor IRRs; the reduced affinity of the TPase for the adjacent B^IRL ^element; and the tendency of the CDs to be bent by the protein. These factors would prevent minicircle formation and therefore define the identity of the recipient end in the wild type element.

In elements with two right ends, however, both function as donors with equal probability and produce minicircles in which approximately 90% of the MCJs have interstitial sequences of 2 bp to 3 bp. This would not be the case if both donor CDs were complexed *in cis *at their cognate CCs (Figure 2A) when the majority of minicircles would have 5 bp interstitial sequences. In complexes formed with two right ends, the CD of one end is bound *in cis *and that of the other bound *in trans*, both at a single active CC. Binding *in trans *would be facilitated by the non-specific and/or selective binding of the adjacent host DNA coupled with the bending of the CD by the protein as indicated by enhancements at residues 9 and 10.

Different conformational states define the protein-DNA interactions of IRR and IRL not only at their outside ends but also at their inside ends, primarily due to the different functions of the ends. At the inside ends of IRR and IRL, different protection patterns involve the two most distal elements (C and III) of the PBD (Figure 6). The stronger protection pattern in elements C^IRL ^and III^IRL ^is a manifestation of the location of the docking site, 5'TAAATAA3', for the repressor function of OrfA, (Figure 1C, ii; [20,40]). The transposase bound to IRL (Figure 5) shows strong protection of the last 4 bp at the inside end of IRL, T/A, T/A, A/T, A/T (residues 39 to 42 of element III), and the 6 bp sequence A/T, T/A, A/T, A/T, G/C, T/A (residues 43 to 48) located immediately adjacent to the inside end and just upstream of P_IRL_, the extended-10 promoter [10]. These two sequences together appear to form a 10 bp sequence which includes the site to which the 14 kDa OrfA binds competitively in carrying out its repressor function. It is interesting that the truncated 17 kDa derivative of the IS*30 *TPase (the structural equivalent of OrfA) has also been shown to overlap the promoter region, likely repressing transcription [37], but that OrfA in IS*911 *does not have this function. Instead, it has been shown to modify the stoichiometry of complexes formed with the 1-149 truncated forms of OrfAB [26]. In addition, in IS*911 *OrfA is involved with both heteromultimerization with OrfAB [41], as well as with its own homomultimerization and with the ability to stimulate minicircle insertion *in vitro *into target DNA not associated with the IS911 ends [28]. It is likely that these heteromultimers may also exist in our preparations, which consist of a mixture of OrfA and OrfAB [31]. Speculatively, in IS*2*, the three-dimensional configuration of OrfAB could allow the BD of the protein to target the B and II elements in the PBD, whereas (as a regulatory mechanism) the BD in OrfA, with a slightly different configuration, would target the promoter.

Three previous studies have reported footprinting analyses of the IS*3 *family and related elements that hint at the bipartite nature of the ends. Earlier, Hu *et al*. [23], using cell-free extracts of the IS*2 *Tpase, reported *in situ *1, 10 phenanthroline-copper ion footprinting data for the bottom strand of the right end (5'-TGG... TTAA-3') and the top strand of the left end (5'-TAG.... TTAA-3') of IS*2*. They showed essentially identical patterns of protection of residues 16 to 41 in the case of IRR and 16 to 42 in the case of IRL with additional protection of residue 43 in the former and protection of residues 43 to 46 in the latter. They reported no binding to the outer base pairs, 1 to 15, for either end, due perhaps to the prevalence of truncated N-terminal species in the preparation of the protein [26] or to the imprecise folding of the C-terminus, a process which appears to have been avoided in our GFP-tagged version [31].

Normand *et al*. [26] reported DNase I and Cu(OP)_2 _(copper-1,10phenanthroline) footprinting data for IRR and IRL single-ends of IS*911 *using a truncated version of OrfAB (residues 1 to 149) from which the carboxy-terminus was deleted; the protein thus consisted primarily of its binding and dimerization domains. Their deletion-gel retardation analyses of the ends of IS*911 *showed that they are composed of three conserved blocks of residues α, β and γ; β and γ comprise the PBD of IRR and IRL whereas the α motif comprises the CD. Footprinting experiments with both IRR and IRL showed that the truncated OrfAB bound efficiently in an extensive manner to the PBDs of the ends. Finally, DNase I footprinting experiments with the 17 kDa N-terminal derivative of the IS*30 *Tpase containing only the BD of the protein, showed binding to the central region of an inner, presumed PBD, leaving the outer termini of both right and left ends unprotected [37].

The bipartite nature of the ends of transposable elements has been well documented by mutational analysis and DNA footprinting studies. The two domains, originally identified through mutational studies in IS*903 *[42], IS*50 *(Tn5) [43,44] and IS*10 *[45], were subsequently shown in early DNA footprinting studies to be a unique inner binding sequence for the transposase and an outer unbound sequence assigned to post binding cleavage functions. This was shown to be true for simple insertion sequences IS*30 *[37], IS*1 *[46], IS*903 *[47], IS*50 *[48] and IS*911 *[26] as well as for the more complex transposons, Tn*3 *[49-51] and Mu [52,53]. Binding to both domains, however, was shown to occur in fully formed SCs in Mu [54-56] and in IS*50 *[36]. We conclude from these analyses that the bipartite binding pattern exhibited in IS*2 *protein/DNA complexes is the result of a fully formed Step I SC.

### Footprinting results in SC I correlate well with those of previous mutational analyses of the PBD of the single IRL end

An earlier mutational analysis of the IRL sequence indicated that, while residues 12 to 19 (primarily the B element) played an important role in protein recognition, an anchoring sequence for the transposase was also located at residues 20 to 42 (elements II, C and III; [24]). In general, the footprinting data (Figure 6) support these conclusions. We assessed the effect of seven single base deletion mutations on the efficiency of minicircle formation and found that there is a good correlation with current binding efficiency data. Deletion of base pairs at positions 13, 19, 21 and 36 had no effect on minicircle efficiency. In these footprinting studies, only position 21 was protected by the protein. Deletions of base pairs at positions 14, 26 and 29 eliminated minicircle formation and only residue 26 was not protected by the protein.

### Footprinting the IS*2 *MCJ

In Step II of the IS*2 *transposition pathway, donor function of each of the abutted ends at the MCJ is a prerequisite for insertion of the element into the target sequence. In an earlier model [10,24], we proposed for the sake of simplicity that the complex involved a dimer of transposase molecules with the PBD of each end bound at its own monomer. Initial cleavage of the abutted CDs of the MCJ would occur at the 3' end of the IRR CD, bound *in cis *at its cognate CC (a first transition state). As a result of a conformational change the partially cleaved junction would be relocated to permit *cis*-binding of the IRL CD at its cognate CC (a second transition state). There, cleavage at its 3' terminus would occur, permitting the reacquisition of *cis *binding by the IRR CD.

To test these ideas, we asked here whether a covalently joined MCJ (substrate MJcj) and a precleaved MCJ (substrate MJpc) would produce different SC II footprinting patterns for the IRR and IRL CDs. The covalently joined MCJ was prepared from two annealed 114 nucleotide oligomers (substrate MJcj in the Methods section) containing an 84 bp sequence of the abutted right and left ends separated by a single guanine/cytosine base pair. For footprinting experiments, the bottom strand (3' to 5') was labeled at its 3' end with alpha ^32^P-labelled di-deoxy adenosine triphosphate ([α^32^P] ddATP). Substrate MJpc (see the Methods section) containing the precleaved MCJ was prepared using a bottom strand identical to that in the MJcj substrate and labeled as described above. The top strand consisted of two oligomers; at the 5'end was a 56 nucleotide oligonucleotide, containing the 41 nucleotide donor strand of IRR ending in its CA-3' terminal dinucleotide. The second component was a 58 nucleotide oligonucleotide containing the 42 nucleotide strand of IRL, with a single nucleotide (C) at its 5' end representing the spacer base between the two abutted ends. The result of the annealing reaction was a double-stranded MCJ with a one base pair spacer, nicked at the CA-3' terminus of the IRR CD. Very efficient binding of the protein to both substrates was observed in EMSA gels (Figure 3B). The slight difference in the running patterns of the two complexes may be attributed to the differences in the structure of the two substrates.

**Figure 7:**
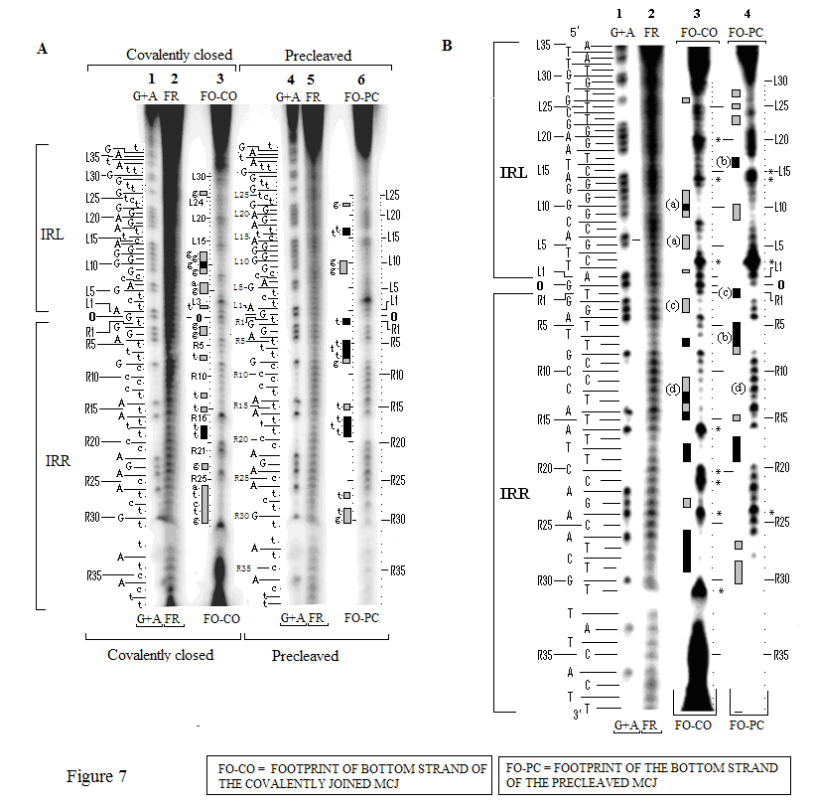
**Footprinting of the bottom strands of the MJcj and MJpc substrates**. Footprinting reactions were run on an 8% polyacrylamide sequencing gel together with the unbound DNA reactions (FR) and the G+A Maxam-Gilbert sequencing reactions (G+A). Vertical grey and black rectangular bars represent weakly and strongly protected residues respectively. The bands in the G+A and footprinted lanes are identified with dots and/or short horizontal lines. The DNA sequence of the bottom strand of the MCJ is shown to the left of the G+A lanes and is numbered as R1 to R39 and L1 to L37 reading from the abutted ends towards to the inside ends of the two termini. The spacer base (G) of the MCJ is numbered as 0. **(A) **Two hour exposure of the gel. **(B) **Overnight exposure of the gel facilitated the ready distinction of weak and tight binding. Bars labeled (a) identify sequences in the CD of IRL that are disengaged in the nicked (MJpc) substrate and more tightly bound in the covalently closed (MJcj) substrate. Bars labeled (b) in the CD of IRR and the PBD of IRL, indicate sequences that are more strongly protected in MJpc than in MJcj. The bars labeled (c) at the terminal trinucleotide of IRR identify differences in binding affinity to this sequence of the two substrates. The (d) labels indicate the loss of binding affinity to the PBD of IRR in the cleaved substrate compared to the covalently joined substrate bringing the protection pattern of the former more in line with that of the single IRR end (see Figure 9). CD: cleavage domain; IRR/IRL: right and left inverted repeats; MJcj: covalently joined minicircle junction substrate; MJpc: precleaved minicircle junction substrate; PBD: protein binding domain.

**Figure 8:**
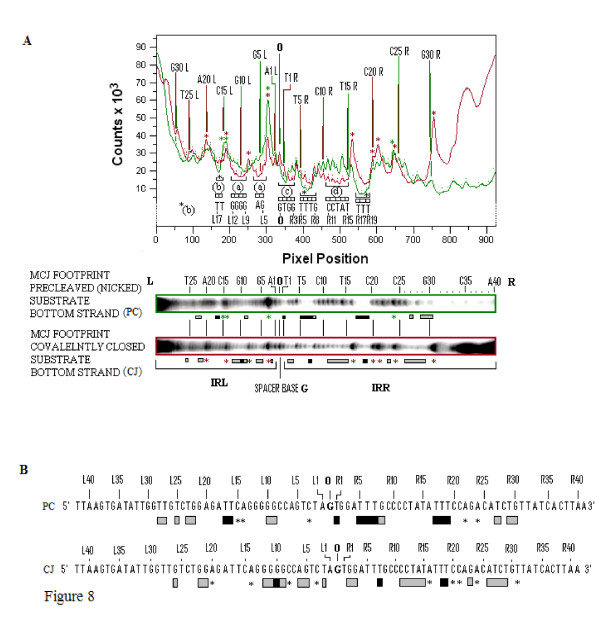
**Quantitative comparisons of the protection patterns of the bottom strands of MJcj and MJpc substrates**. **(A) **The top panel shows densitometer tracings of the two footprinted lanes (MJcj, red, and MJpc, green) of the sequencing gel shown in Figure 7b. The similarly color-coded boxed lanes are shown immediately below the panel. Tracings show differences in the intensities of bands from the two substrates. Annotation within the panel is based on the sequence of the bottom strand with numbering as described in Figure 7. Individual peaks in the top panel are identified by red dots for the covalently joined substrate and green dots for the nicked substrate; corresponding red and green vertical lines identify the nature and number of every fifth base. Differences in the protection patterns of the two substrates are indicated by brackets (within which the protected residues are identified) immediately beneath the troughs. Labels (a), (c) and (d) are as described in Figure 7. Brackets labeled with a black asterisk or (b) indicate sequences that are more strongly protected in MJpc than in MJcj. Enhanced residues in the two substrates are shown by sharply rising peaks and are identified by the eight red asterisks for the MJcj substrate and the four green asterisks for the MJpc substrate. **(B) **Consensus of the protection patterns of the bottom strand of the MJcj and MJpc substrates are derived from the data in Figures 7A, B and Figure 8A. Numbering and annotations are as described in Figure 7. Asterisks identify enhanced residues. MJcj: covalently joined minicircle junction substrate; MJpc: precleaved minicircle junction substrate.

Footprinting patterns for the bottom strands of the two 114 nucleotide MCJ substrates are shown in Figure 7A. Side-by-side lanes of the G+A Maxam-Gilbert reactions, the two cleaved unbound controls and the footprinted covalently closed and precleaved substrates, are shown. Each bottom strand is numbered as R1 to R41 and L1 to L42 reading from the abutted ends outwards. The spacer base guanine is numbered as zero. A larger, higher contrast version of the same gel which accentuates the protected residues is shown in Figure 7B. Comparative densitometer tracings for the precleaved and covalently joined MCJ substrates from the gel in Figure 7 are shown in Figure 8A their protection patterns are described in Figure 8B. Because of the length of these substrates, data for the nine bases at the inside ends of IRR and IRL (that is, resides 33 to 42) were difficult to ascertain and are excluded from this analysis.

### CDs in the MJcj substrate are complexed to the same catalytic center followed by a cleavage-triggered conformational change

**Figure 9:**
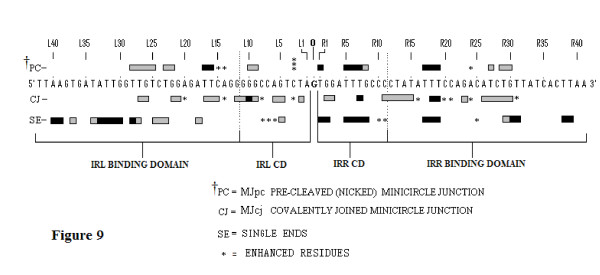
**Comparisons of protection patterns of the bottom strands of the single-end, MJcj and MJpc substrates**. The sequence shown is that of the bottom strand of the MCJ with the spacer base guanine separating the abutted right and left ends. Numbering of the bases is as described in Figure 7. Protection patterns are indicated by horizontal bars. Asterisks identify enhanced residues. Three stacked asterisks describe increased enhancement. Broken vertical lines within the large brackets demarcate the IRR and IRL cleavage domains (CD) and protein binding domains (PBD). Data for residues L30 to L42 and R32 to R41 of the MCJ substrates were difficult to interpret and are not shown. CD: cleavage domain; IRR/IRL: right and left inverted repeats; MCJ: minicircle junction; MJcj: covalently joined minicircle junction substrate; MJpc: precleaved minicircle junction substrate; PBD: protein binding domain.

**Figure 10:**
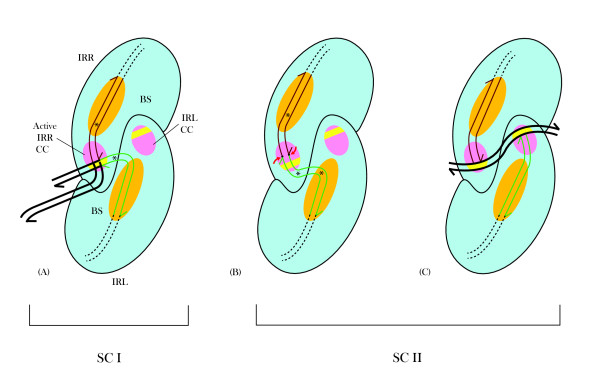
**Schematic model for the IS*2 *transposition pathway**. Each synaptic complex is shown as a dimer with a DNA binding site (BS; orange) to which protein binding domains (PBD) of the right and left inverted repeats (IRR, red and IRL, green) are bound, and a catalytic center (CC; pink). Each CC possesses a binding tract (orientation I), for the extensive sequence-specific binding of the cleavage domain (CD) and a tract (yellow band; orientation II), at which target or host DNA may be complexed selectively and/or non-specifically. **(A) **Synaptic Complex I. The CD of IRR is bound in orientation I *in cis *at its cognate active CC. The IRL CD is bent (asterisk) and complexed at the active CC *in trans *in orientation II, with adjacent host DNA (bold black lines). The 3'OH tip of the cleaved IRR CD is positioned at a 3' 5' phosphodiester bond between the first and second residues of the host DNA near the 5' end of the tip of IRL. Broken black lines represent the coding sequence of IS*2*. **(B) **Synaptic Complex II- first phase. Abutted CDs of the MCJ separated by a single base pair spacer (bold black dot), are bound in orientation I at the active IRR CC. *Trans *binding of the IRL CD is facilitated by two bends (asterisks), within the CD and at the outer end of the PBD. Red arrows identify sequential single-strand cleavages at the 3' ends of the CDs. **(C) **Synaptic Complex II- second phase. The CD of IRL, free from flanking DNA, binds to its cognate CC in orientation I. Exposed 3'OH groups at the ends of both CDs (half arrows) are juxtaposed to the target DNA, non-specifically bound (curved bold black lines) in the orientation II tracts of the CCs. BS: binding site; CC: catalytic center; CD: cleavage domain; IRR/IRL: right and left inverted repeats; MCJ: minicircle junction; PBD: protein binding domain.

Several features help compare and contrast the protection patterns of the covalently joined and precleaved substrates. We can also contrast these with the protection patterns of the single-end substrates. Comparative schematic representations of the protection patterns of the bottom strands of the four substrates, that is, the two single-end substrates and the two MCJ substrates, are shown in Figure 9. First, some residues within two short sequences (R1 to 3 and 5 to 8) in the bottom strand of the IRR CD are protected in all three substrates (compare residues R1 to R8 in Figure 9). The similarity of protection patterns is particularly true for the MJpc and single-end substrates. The lower affinity for the residues in the MJcj IRR CD may be a consequence of the need to accommodate binding and cleavage of the IRL CD post-IRR cleavage at the same active CC, implying that cleavages are sequential and that the cleavage of IRL occurs *in trans*. We thus conclude that the IRR CD is bound *in cis *at its cognate CC in all three substrates (Figure 10A, B).

Secondly, in the MJcj substrate, the CDs of both IRL and IRR are protected in a similar manner by the protein (compare L2, L5, L6 and L9 to 11 with R2, R3, R7 and R11 in Figure 9 and 7B, lane 3). This result suggests that the CD of IRL in the MJcj substrate is extensively bound at the same CC as the IRR CD. Since the two CDs in the IS*2 *MJcj substrate are separated by a single base pair, their observed extensive protection (summarized in Figure 9) should result from initial binding of both CDs (the IRL *in trans *and the IRR *in cis*) at a single active CC (Figure 10B). This represents the first phase of the SC II complex in IS*2*. A similar scenario may apply to IS*21 *[29], IS*1665 *[30] and IS*256 *in Tn*4001 *[28].

Thirdly, the MJpc substrate shows evidence of disengagement of the IRL CD from the TPase (Figure 9). In the covalently joined substrate, three sets of residues within the IRL CD are bound moderately tightly (L2 (T), L5 and L6 (GA) and L9 to L12 (GGGG)); of these, only two residues (L9 and L10) are protected in the precleaved substrate (see also the protection patterns labeled (a) in Figures 7B and 8A). This is not the case for the IRR CD where binding is even more extensive than in the MJcj. Based on two lines of evidence, we conclude that the partially cleaved junction is not positioned at the IRL CC after right end cleavage, as suggested in our original hypothesis. First, the apparent disengagement of the IRL CD suggests that it is not bound extensively at its cognate CC. Secondly, the IRR CD in the precleaved substrate remains bound at its cognate CC as judged by the similarity of its protection pattern to that of the single-end substrate. We propose then, that the protection patterns observed for the MJpc substrate represent those of a temporary (and artifactual) transition state and that complete disengagement of the IRL CD would follow the two sequential single-strand cleavages at the IRR CC (Figure 10B). After a conformational change, re-engagement of the IRL CD at a new site, its cognate CC, would then occur to produce a second complex in SC II (Figure 10C).

There is additional evidence for this temporary transition state. Two differences within the CDs of the two MCJ substrates at residues R1 (T) and R5 to R8 (TTTC) make the profile of the IRR CD in the MJpc substrate almost identical to that of the single-end substrate (Figure 9; see also the gel in Figure 7B, lane 4, protection patterns labeled (b) and (c)). Also, there are subtle differences in the protection patterns of the IRR PBD in the two MCJ substrates; residues R11 to R15, which are protected in the MJcj substrate, are disengaged in the MJpc substrate (Figure 7B, compare lanes 3 and 4, protection patterns labeled (d)), again making its protection pattern almost entirely like that of the single-end substrate (Figure 9). We note that major changes in protection patterns do not affect the PBDs. There is a basic similarity but not identity in the protection patterns within the PBDs IRR (R17 to R19 and R26 to R31) and IRL (L15 to L18 and L21 to L28) in each of the three substrates (Figure 9).

It is now well understood that the process of transpositional recombination is controlled by a series of conformational changes within the transpososome that drive the process forward unidirectionally. These may be triggered by cleavages [57], host proteins [58], divalent cations [59], the role of terminal cognate nucleotides [60] and associated transposition proteins [61]. It appears here that sequential cleavages at the abutted IRR and IRL CDs of the first phase in the SC II transpososome of IS*2 *would provoke the conformational change that is required for the establishment of the second phase that is needed for final strand transfer reactions into the target DNA.

### The sequence of the IRL CD has evolved to permit selective binding in SC I without compromising extensive sequence-specific binding in SC II

In earlier studies we proposed that the non-conserved base pairs in IRL were necessary for efficient recipient end function and were sufficient to prevent binding of the CD *in cis *to its cognate active site in SC I, without compromising binding proficiency in SC II [24]. Footprinting data for the MJcj substrate support these suppositions. Six of the eleven residues within IRL CD in the bottom strand of the MJcj substrate are bound by the protein. The non-conserved residues at positions L2 and L5 are protected, as is the run of guanines at positions L9 (non-conserved) to L12 (Figure 9). Protection of this guanine/cytosine run is characteristic of strong extensive binding in the single-end IRR substrate and is not observed in the single-end IRL substrate (Figure 6). Thus, two of the three residues in the IRL CD that are involved in selective binding in SC I, are also utilized in extensive sequence-specific binding in SC II. It seems likely that this extensive sequence-specific binding of the IRL CD in the MJcj substrate results partially from its proximity to the extensively bound IRR CD. In addition, given the proximity of selective binding of the IRL CD and the non-specific and/or selective binding of the adjacent host DNA in the L79 single-end substrate (Figure 6), we propose that the nature of (or the absence of) the DNA adjacent to the cleavage domain of IRL plays a decisive role in determining whether it is involved in selective binding or extensive sequence-specific binding.

### Evidence for bending of the DNA in MCJ and single-end substrates from footprinting data is corroborated by curvature propensity plot data from the MJcj sequence

The differences in the protection patterns of the MJcj and MJpc may be due to perturbation, generated as suggested above by the binding of both CDs at a single active site. Perturbation in the MJcj substrate is indicated by the presence of five enhanced residues in the PBD of IRR and four enhanced residues in the CD and the PBD of IRL. In contrast, in the MJpc substrate, only a single residue (R24) is enhanced in the PBD of IRR (Figure 9; see also Figure 7B, lanes 3 and 4 and Figure 8), suggesting not only that perturbation of the IRR DNA is relieved following cleavage but that the remaining enhanced R24 residue in both substrates is indicative of intrinsic bending at that site. In addition, the location of the enhanced region within the PBD of the IRR single end substrate (see residues R25 and R29 to R30; Figure 6) is similar to that in the two MCJ substrates (see residue R24; Figure 9), suggesting a single bend in IRR DNA in all three substrates. In a similar vein, enhanced residues in IRL are observed at two identical locations in the MJcj and MJpc substrates, that is, residue L3 in the CD and L14 at the outer end of the PBD (Figures 8 and 9). An enhanced residue is also observed at L15 in MJpc. In addition the enhanced region in the CD of the IRL single-end substrate (see residues L1, L3, L6 to L8 and L10; Figure 6) is in the same location in the MCJ substrates (see residues L3 and L8; Figure 9), suggesting that the tip of IRL is bent in all three substrates to accommodate binding *in trans *to its CD at the active IRR CC as illustrated in Figure 10A, B. Thus the presence of enhanced residues that are at common or near common locations in the single end, MJcj and MJpc substrates may be indicative of bending at intrinsically bent sites (see below).

**Figure 11:**
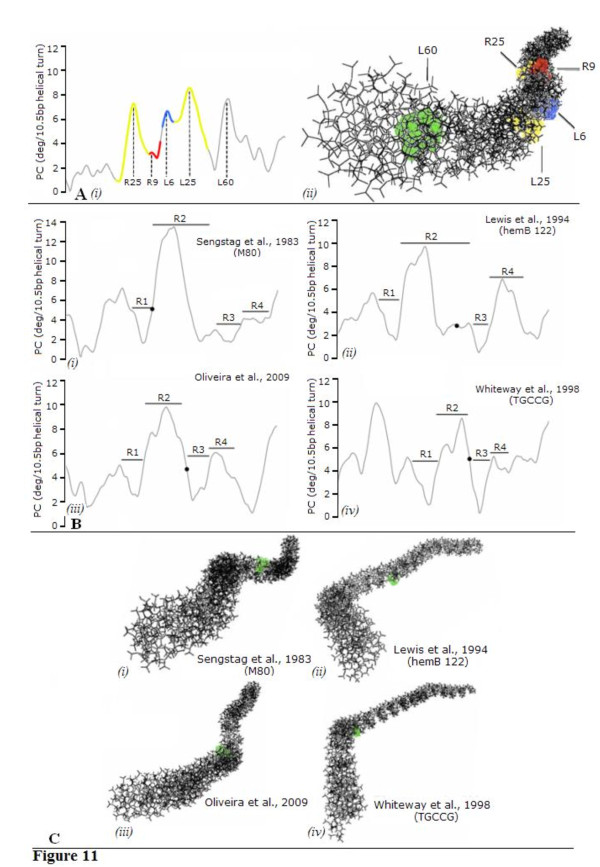
**Curvature analyses for the minicircle junction and IS*2 *target sites**. **(A) **(*i *) Predicted curvature profiles obtained by the bend.it server for a 200-bp region encompassing the MCJ. Colored regions are: IRR (yellow and red), IRL (blue and yellow), protein binding domains (yellow), cleavage domains (red and blue). Numbered base pairs correspond to the four maxima found in these regions, which also match or are located in close vicinity to enhanced residues. The maximum located at position L60 corresponds to the region harboring the indigenous P_IRL _promoter. (*ii *) Three-dimensional representation of the region encompassing the MCJ where the five curvature maxima appear as highlighted bases. The region shaded green represents the intrinsic curvature of the P_IRL _promoter. **(B) **Predicted curvature profiles of four representative regions reported in the literature to harbor IS*2 *target sites. Each window represents a 200-bp fragment encompassing the target site(s) (filled circles). Regions R1 to R4 were arbitrarily chosen in order to facilitate the comparison between graphs. Although some disparity exists when comparing the relative intensity of the peaks (which results from comparing different DNA sequences), all four regions appear to be conserved. Coding references or nucleotide sequences given in brackets are in accordance with the nomenclature given in the original publication. Additional predicted curvature profiles are shown in Additional file 3. **(C) **Three-dimensional representations of the four regions encompassing IS*2 *target sites (highlighted in green). S-like (and L-like) shaped regions were preferentially obtained and intrinsic curvature was observed to occur next to the insertion site. Additional data on the three-dimensional representation of IS*2 *target sites can be found as Additional file 4. bp: base pair; IRR/IRL: right and left inverted repeats; MCJ: minicircle junction.

The observation that these sequences, which are consistently bent at approximately the same positions in SC I and SC II, occur at regions associated with guanine/cytosine-rich tracts in the CDs prompted us to evaluate their intrinsic curvature (that is, the permanent or time-averaged deflexion of the DNA axis when no external force is applied) by analyzing the abutted terminal repeats of IS*2 *with the bend.it server (see Methods). The purpose of using this tool was to evaluate whether regions are inherently curved during the interactions between CDs within the SC II complex. According to the curvature propensity plot obtained (Figure 11A (*i*)), three strong maxima are evident in the IRR and IRL regions of the MJcj sequence: two in the PBDs (R25, L25) and one in the IRL CD (L6). In addition, a weak maximum is observed in the IRR cleavage domain at R9. With the exception of L25 in the IRL PBD, the remaining positions match or are located close to the enhanced residues in the footprinting gels (compare with Figure 9) but the position of L25 still appears to be related to the enhancement data in the MCJ substrates, in that both sets of data suggest that there is a bend at the outer half of the PBD of IRL. A fifth maximum is also present at L60 which corresponds to the location of the indigenous P_IRL _promoter. This is expected, for promoter sequences are well known to be characterized by intrinsically curved DNA [62,63]. To better understand exactly how this curvature profile translates in terms of DNA architecture, we generated a three-dimensional representation (Figure 11A (*ii*)) using the same MCJ DNA and obtained an S-like structure which is typical of some regional anisotropic flexibility. As stated above, this seems to result from an overrepresentation of guanine/cytosine-rich tracts in the CDs, which, in conjunction with other properly phased sequences, results in a preferential curvature.

We have asked whether the curvature maxima identified within the MJcj sequence are a reflection of the intrinsic curvature resulting from the interaction of the two CDs or curvature associated with the powerful promoter within the MCJ sequence. Since the curvature maxima at L6 and R25 correspond to enhancements in both the single-end substrates and the MCJ substrates as described above, we interpret the enhancements in the L79 (IRL) substrate as the result of bending to accommodate binding of both IRL CDs *in trans *in SC I (see the bend in IRL in Figure 10A) and in the MJcj substrate as bending to accommodate binding of the abutted CDs of the MCJ to a single CC in SC II (Figure 10B); indeed, observed perturbations of the DNA in the footprinted gels of the MJcj and MJpc substrates or of the single ends may result from the DNA being bent by the protein in the same direction as the intrinsic sequence-dependent curvature [64]. We note that there is no enhancement of the residues in the PBD of the L79 (IRL) single-end substrate corresponding to the L25 maximum of the MCJ sequence (see below). In addition, the weak curvature maximum at R9 in the MJcj sequence is probably a relic of the type of IRR CD to CD interaction described earlier, for elements with two right ends. Thus the intrinsic curvature data not only corroborate the footprinting data but also support the idea that interaction of the CDs at a single active CC requires the adoption of a bent structure.

### Intrinsic curvature of IS*2 *target sites

Because binding of the IRL CD *in trans *at the active CC within SC I and SC II seems to require the DNA to adopt a bent structure, we wondered whether IS*2 *target sites could also be structurally constrained. Therefore we decided to look at the predicted curvature of 200 bp-sized sequences from several IS*2 *target sites reported in the literature. A representative sample of these is shown in Figure 11B (the remaining curvature profiles are presented in Additional file 3). An interesting feature of the data is a consistent periodic behavior of the predicted curvature of sequences flanking the target sites. Subsequent analysis led to the division of the target sites into four regions (R1 to R4), a profile which was roughly similar in all of the DNA sequences examined. R1 holds a local minimum in predicted curvature, R2 a local maximum harboring a shoulder peak that sometimes appears as two well resolved peaks and regions, and R3 and R4 hold a local minimum and maximum respectively. IS*2 *insertion sites (black dots) mapped preferentially within the sub-sequences of R2 with a mean curvature of 4.4 ± 1.9 degrees per 10.5 bp helical turn. It is thus tempting to assume that the choice of insertion site might depend on DNA curvature at the target with the decision for integration based on subsequences of R2 having a certain range of curvature values. This similarity between curvature profiles is reflected in the three-dimensional structure of each region (Figure 11C) where an S-like (and sometimes L-like) structure is preferentially adopted. IS*2 *insertion sites were found to be located between two bent regions ([65,66]; Figure 11C, i, ii) or alternatively exactly at a bent region ([67,68]; Figure 11C, iii, iv). Additional three-dimensional representations of the curvature profiles are also presented in Additional file 4.

### A model for the two-step transposition pathway of IS*2*; CD to CD interactions require that IRL adopt a bent structure in SC I and SC II

We describe here a refined version of our model for SC I and SC II [24]. For SC I, single bends of the IRR PBD and of the IRL CD are required to synapse the CDs in two different orientations, I and II respectively, at the single active CC as illustrated in Figure 10A. For the first phase of the SC II complex, binding of the two CDs separated by a single base pair suggest that the CDs are complexed in orientation I at the active IRR CC. Two bends of IRL, at the CD and the outer end of the PBD and a single bend of the IRR PBD are needed to achieve this binding arrangement (Figure 10B), where sequential cleavage reactions would occur to generate the second phase of the complex (Figure 10C).

Intrinsic curvature data have indicated that both MCJ DNA and target sites adopt bent structures that apparently share identical profiles (compare Figure 11A (*i*) and 11B). Given the large number of target sites analyzed (Additional file 4), it is tempting to assume that curving propensity might play some role in target site selection although it is not clear how and to what extent this would affect the mechanics of transposition. A similar dependence between transposition and target curvature has been shown to exist for IS*231 *[38], where target sites contain alternate guanine/cytosine- and adenine/thymine-rich tracts that promote bending in opposite directions of the regions flanking the consensus target sequence. In a more recent example, Kobori *et al*. [69] reported a target site for the spontaneous insertion of IS*10 *located within an intrinsically bent DNA region of the commonly used vector pUC19. Likewise, we observe from Figure 11C that IS*2 *preferentially inserts in the close vicinity of curved regions or specifically at a bent region. This concept has been incorporated into the model of the second phase of the SC II complex, where curved target DNA is now bound non-specifically across each CC permitting strand transfer to the target by each donor end (Figure 10C).

## Methods

### Bacterial strains and media

*Escherichia coli *strain JM105 was used for cloning and for most procedures involving plasmid DNA preparation. DNA transformation was carried out into supercompetent XL1 Blue cells (Stratagene Inc., Santa Clara, CA, USA) for reactions requiring cloning and expression of pLL2522, the plasmid with the fused *orfAB *and *GFPuv *genes.

Cultures were routinely grown in lysogeny broth media at 37°C, supplemented where necessary with carbenicillin (Cb, 50 μg/mL) or chloramphenicol (Cm, 20 μg/mL). For the overexpression of the fused *orfAB::GFP *genes in plasmid pLL2522, cultures were grown at 28°C in a 2× yeast extract and tryptone (2 × YT) medium supplemented with Cm, Cb and arabinose (6 mg/mL).

### DNA procedures

DNA procedures were essentially as described earlier [10,11,24].

### Plasmid constructs

pLL2522, which contained the fused *orfAB *and *GFPuv *genes, has been described in detail previously [31].

### Preparation of the OrfAB-GFP fusion protein under native conditions

Plasmid pLL2522 was transformed into BL21(DE3)pLysS cells (Stratagene Inc.). Single colonies were inoculated into 40.0 mL of 2 × YT medium supplemented with Cm, Cb and arabinose and inoculated in baffled flasks overnight at 28°C. Harvested pellets were checked for bright fluorescence, washed with 3.0 mL Native Wash Buffer (Qiagen, Valencia, CA, USA) and frozen at -70°C for 15 min. Three milliliters of B-PER Protein Extraction Reagent (Thermo Scientific, Pierce Protein Research Products, Rockford, IL, USA), supplemented with 4.0 μL of Benzonase (Novagen-EMD4Biosciences, La Jolla, CA, USA), per 40 mL of overexpressed culture and 3.0 mL Protease Arrest (Calbiochem/EMD La Jolla, CA, USA) per milliliter of lysate was added to the frozen pellet, which was allowed to thaw on ice on a horizontal rotary shaker for 60 min. The lysate was nutated at 4°C for 1 h and subjected to a hard spin at 10,000 ×g for 45 min at 4°C. It was then purified through Ni-NTA His-tag technology. 6 × His-tag purification of the protein was achieved by gravity flow affinity chromatography using Ni-NTA agarose (Qiagen) under native conditions essentially following the manufacturer's instructions. The crude lysate was loaded on to a 1.0 mL bed of the nickel-charged resin in a 5.0 mL column for chromatographic separation followed with UV light. The protein bound as a tight brightly fluorescing band at the top of the column and remained bound through washings with 10 mM to 60 mM imidazole, when a slight dissociation of the band was observed. To circumvent continued dissociation, the band was eluted with 250 mM imidazole and its progress through the column followed. Peak fractions (fluorometrically determined) were subjected to diagnostic 12% PAGE using acrylamide and bis-acrylamide (Ac:Bis; 30%:8%, respectively) polyacrylamide gels [31]. Fractions showing both the 74-kDa OrfAB::GFP and the 17-kDa OrfA proteins were pooled (approximately 700 μL), concentrated to about 75 μL in a YM-10 Microcon Centrifugal Filter Device (Millipore, Billerica, MA, USA), dialyzed overnight in Slide-A-Lyzer cassettes (Thermo Scientific, Pierce Protein Research Products) and stored in 50% glycerol at -20°C. The concentration of the fused OrfAB-GFP protein was measured with spectrophotometry at 397 nm and that of the control GFP at 280 nm and 397 nm. Comparative levels of fluorescence of GFP and the fusion proteins were measured with fluorometry and used to confirm the concentration data.

### Oligonucleotides used in gel retardation and DNA footprinting experiments

The right single end (IRR) was represented by an 87-bp substrate R87. The IRR sequence is shown between the brackets. Top strands were labeled at the 5' end and bottom strands at the 3' end. The top strand (primer A) sequence was as follows: 5'GCTGACTTGACGGGACGGGGATCC[TTAAGTGATAACAGATGTCTGGAAATATAGGGGCAAATCCA]ATCGACCTGCAGGCATATAAGC3'; the bottom strand (primer B) sequence was as follows: 5'GCTTATATGCCTGCAGGTCGAT[TGGATTTGCCCCTATATTTCCAGACATCTGTTATCACTTAA]GGATCCCCGTCCCGTCAAGTCAGC3'.

The left single end (IRL) was represented by the 78-bp substrate L79. The IRL sequence is shown between the brackets. The top strand sequence (primer A) was as follows: 5'ACGCGGAGTGAATTCGAGCTC[TAGACTGGCCCCCTGAATCTCCAGACAACCAATATCACTTAA]ATAAGTGATAGTCTTA3'; bottom strand (primer B) sequence was as follows: 5'TAAGACTATCACTTAT[TTAAGTGATATTGGTTGTCTGAAGATTCAGGGGGCCAGTCTA]GAGCTCGAATTCCACTCCGCGT3'.

The covalently closed MCJ was represented by the 114-bp substrate, MJcj. The abutted IRR (bold) and IRL sequences, shown in the brackets, are separated by a single base pair guanine/cytosine spacer. The top strand sequence (primer A) was as follows: 5'GGTACCCGGCCATGG[**ttaagtgataacagatgtctgggaaatataggggcaaatcca**]C[TAGACTGGCCCCCTGAATCTCCAGACAACCAATATCACTTAA]ATAAGTTATAGTCTT3'; bottom strand (primer B) sequence was as follows: 5'AAGACTATAACTTAT[TTAAGTGATATTGGTTGTCTGGAGATTCAGGGGGCCAGTCTA]G[**TGGATTTGCCCCTATATTTCCAGACATCTGTTATCACTTAA**]GGATCCCCGGGTACC3'.

The precleaved (nicked) MCJ was represented by the 114-bp MJpc substrate. Two oligonucleotides were needed to create the top strand. The first, a 56-mer oligonucleotide contained the IRR sequence (bold font) terminated with an A-3'OH at the junction and was labeled at its 5' end. The sequence for the top strand (primer A1) was as follows: 5'GGTACCCGGGGATCC[**TTAAGTGATAACAGATGTCTGGAAATATAGGGGCAAATCCA**]3'.

The second primer, a 58-mer oligonucleotide, terminated at its 5' end with a cytosine representing the single spacer nucleotide. It was labeled at its 5' end. Its sequence (Primer A2) was: 5'C[TAGACTGGCCCCCTGAATCTCCAGACAACCAATATCACTTAA]ATAAGTTATAGTCTT3'. The bottom strand was identical to that described for the MJcj substrate.

### 5'- and 3'- end labeling and annealing of the oligonucleotides

**5'-end labeling of the primers: **A 20-μL labeling reaction contained 30 units of T4 polynucleotide kinase (New England Biolabs, Ipswich, MA, USA), 2.0 μL of 10X T4 polynucleotide kinase reaction buffer, 20 μM of the primer, 50 μCi of the gamma ^32^P-labeled adenosine triphosphate (γ^32^PATP) (6000 Ci/mmole). The reaction was incubated at 37°C for 30 min and heat killed at 90°C for 5 min.

**3'-end labeling of the primers: **The 50-μL reaction contained 20 units of terminal transferase in 1X reaction buffer (USB Corp, Cleveland, OH, USA), 20 μM of the oligonucleotide and 50 μCi of α^32^PddATP. The reaction was incubated at 37°C for 1 h, terminated with 10 μL 2 M ethylenediaminetetraacetic acid (EDTA) and heat killed at 70°C for 10 min.

A 100-μL annealing reaction contained 10 ρmol and 13 ρmol of the labeled and unlabeled strands respectively, 20 mM tris(hydroxymethyl)aminomethane-chloride (Tris-Cl) pH 8.0, and 100 mM sodium chloride. The reaction was placed in a boiling water bath, cooled to 65°C, held there for 15 min and allowed to cool to room temperature. Annealed oligonucleotides were stored at -20°C.

### Protein-DNA complex formation and EMSA

Protein-DNA binding reactions were carried out in 20-μL reaction mixtures with 20 mM Tris-Cl, pH 8.0.Cl, 1 mM EDTA, 1.0 μg/mL calf thymus DNA, 2 nM of the radioactively labeled annealed primers and 80 nM of the partially purified preparation of the OrfAB-GFP fusion protein. Reactions were incubated for 30 min at room temperature and electrophoresed through 5% 19:1 Ac:Bis native polyacrylamide gels at 4°C for 1,000 Vhr.

### In-gel cleavage assays of OrfAB complexed with IRR substrates

**DNA substrates used in complex formation**: An 87-bp IRR substrate (see description of oligonucleotides) and a 50-bp IRR substrate [31] were used in the preparation of protein-DNA complexes. Three types of complexes were formed: (a) with the 50-bp substrate alone, (b) with the 87-bp substrate alone and (c) with a mixture of the 50-bp and 87-bp substrates. Complexes were electrophoresed as described above.

**In-gel excision of the complexes and activation of the TPnase: **Complexes were excised and activation effected based partly on the protocol of Bhasin *et al*. [36]. Essentially the gel was wrapped and exposed to X-ray film for 30 min. It was then superimposed over the developed film and complexes excised based on the location of the images. Each excised gel slice was cut in half and placed into separate 2.0-mL eppendorf tubes. To one tube, 1 mL of an activation buffer (20 mM 4-(2-hydroxyethyl)-1-piperazineethanesulfonic acid, 100 mM K glutamate and 10 mM magnesium chloride or magnesium acetate) was added. To the second control tube, 1 mL of the same buffer lacking Mg^++ ^was added. Gels were incubated at 37°C for 5 min and rinsed twice with 1.0 mL nuclease free water (Ambion/Life Technologies, Grand Island, NY, USA).

**Elution of DNA from gel slices: **The gels were crushed with a micro pestle in 1.0 mL of a 'crush and soak' buffer (10 mM Tris.Cl, 1% SDS and 10 mM EDTA) and nutated at 4°C overnight. The gel pieces were pelleted at 14 K rpm in a microcentrifuge at room temperature for 10 min then rinsed in 500 μL of the same buffer. The resulting 1.5 mL supernatant was then reduced to about 400 μl with three consecutive 14 K rpm spins in a YM-10 Microcon Centrifugal Filter Device (Millipore). Each sample was then subjected to seven buffer exchange (topped up with 400 mL Tris-EDTA pH 8.0) spins at 14 K rpm for 16 min at room temperature. Samples were dried down to a pellet in a Savant SpeedVac DNA concentrator (Savant Instruments, Inc., Holbrook, New York, USA) and resuspended in 2.5 μL nuclease-free water, 2.5 μL of gel loading buffer (GE Healthcare Biosciences, Piscataway, NJ, USA), placed in a boiling water bath for 5 min and stored at -20°C.

**DNA sequencing reactions: **These were carried out essentially as described previously [10,11,24].

### Hydroxyl radical footprinting protocols

Two reactions, one for the footprinting experiment and the other for the free DNA control, were prepared for each substrate as described for the EMSA protocol but with two modifications. Hydroxyl radicals were generated by the Fenton reaction [70]. Reactions were carried out in 70-μL volumes and protein was added to the footprinting tube only, at a final concentration of 225.7 nM. The tubes were incubated at room temperature for 30 min and then subjected to OH radical cleavage. Final concentrations of 5 mM ferrous ammonium sulfate ((NH_4_)_2 _Fe(SO_4_)_2_.6H_2_O), 10 mM EDTA and 0.05% hydrogen peroxide were added to each tube to bring the final volume to 100 μL. These reactants were added as three drops to the side of the tube, then mixed and immediately combined with the sample. The reaction was incubated at room temperature for 2 min and stopped by adding an equal volume of stop buffer consisting of 4% glycerol, 0.6 mM sodium acetate (NaOAc)and 50 μg/mL tRNA. Thiourea was also added as a stop reagent to a final concentration of 11.4 mM.

Purification of the DNA was initiated by removing the protein by the addition of an equal volume of phenol-chloroform-isoamyl alcohol (25:24:1; Sigma-Aldrich, St. Louis, MO, USA), vortexing for 10 s and centrifuging at 15,000 ×g for 2 min. Aqueous layers were removed from each of two repetitions and the DNA was precipitated by adding first NaOAc and glycogen to final concentrations of 100 mM and 0.3 μg/mL, respectively, and then twice the reaction volume of 100% ethanol kept at -20°C. The reaction was stored at -70°C overnight and pellet recovery followed standard procedures [71]. The pellet was dissolved in 10 μL formamide-based loading buffer and stored at -20°C. G+A Maxam-Gilbert sequencing reactions followed the standard procedure [71]. The three reactions, footprinting, free DNA and Maxam-Gilbert, were run side by side in 8.0% polyacrylamide sequencing gels at 1400 v 40 W. The results were quantified on a Typhoon phosphorimager 9400 (GE Healthcare).

### *In silico *prediction of intrinsic DNA curvature

Curvature propensity plots were obtained using the BEND algorithm [72] by submission of DNA sequences to the bend.it server (http://hydra.icgeb.trieste.it/dna/bend_it.html; [73]) using the DNAse I-based parameters of Brukner *et al*. [74]. This server calculates DNA curvature as a vector sum of dinucleotide geometries (roll, tilt and twist angles) and expresses it as degrees per helical turn (10.5° per helical turn = 1° per base pair). DNA sequences were submitted in raw format and the predicted curvature was collected through email in ASCII format. Three-dimensional representation of the curvature profiles was performed with the model.it server (http://hydra.icgeb.trieste.it/dna/model_it.html; [73]) and the output was displayed and visualized with MOLEGRO Molecular Viewer http://www.molegro.com/mmv-product.php. A literature search was performed to analyze the intrinsic curvature of IS*2 *target sites and a detailed list of several DNA sequences from genomic, phage and plasmid DNA encompassing different IS*2 *target sites was gathered. Each of these sequences was analyzed in 200 bp-sized windows by bend.it and model.it. The mean curvature of all IS*2 *target sites was also computed.

## Abbreviations

Ac: acrylamide; Bis: bis-acrylamide; bp: base pair; BD: binding domain; BS: binding site; cb: carbenicillin; CC: catalytic center; CD: cleavage domain; cm: chloramphenicol; EDTA: ethylenediaminetetraacetic acid; EMSA: electrophoretic mobility shift assay; F-8: figure-of-eight; GFP: green fluorescent protein; IRR/IRL: right and left imperfect, inverted repeats; IS: insertion sequence; kb: kilobases; kDA: kiloDaltons; MCJ: minicircle junction; MJcj: covalently joined minicircle junction substrate; MJpc: precleaved minicircle junction substrate; NaOAc: sodium acetate; Ni-NTA: nickel-nitrilotriacetic acid; orf: open reading frame; PBD: protein binding domain; SC: synaptic complex; TPase: transposase; tris-Cl: tris(hydroxymethyl)aminomethane-chloride; 2 × YT: 2× yeast extract and tryptone.

## Competing interests

The authors declare that they have no competing interests.

## Authors' contributions

PTU designed and produced the fusion construct. PTU and RS developed and carried out protein purification protocols. LAL developed the protocols for and carried out the footprinting experiments. SA and JA assisted with and carried out some of the footprinting experiments. LAL designed the study and wrote the manuscript. MA assisted in the experimental design and the writing of the manuscript. LAL provided funding and facilities in New York. PHO and GAM designed and carried out the propensity plot and curvature analysis experiments. DMFP provided funding and facilities for the propensity plot and curvature analysis experiments in Lisbon. All authors read and approved the final manuscript.

## Supplementary Material

Additional file 1 **Activity of the OrfAB-GFP fusion protein in cleavage assays with IRR substrates. (A) **Schematic of expected complexes and ^32^P-labeled single-strand products from mixtures of double-stranded 87 bp (see description of oligonucleotides) and 50 bp [31] IRR substrates and the OrfAB-GFP protein. The 114 nt and 96 nt products would confirm the formation of paired-end complexes (PEC) and the cleavage and joining reactions of SC I (Figure 1a). For simplicity only interactions of "donor", 5' --- > CA3', and "target", 5'TG ---- > 3', strands are shown. The 87 bp substrate was labeled at the 5' end of the "target" strand and the 50 bp substrate at the 5' end of the "donor" strand. "Host DNA" sequences of 22 bp and 3 bp flanked IRR at its outside end in the 87 bp and 50 bp substrates respectively. Three possible PECs (*i-iii*; dimers of red spheres) and their cleavage outcomes are illustrated. The curved arrow depicts the cleaved donor strand and its transesterification attack on the target strand. Recombinant products are only predicted when two 47 nt strands from 50 bp substrates are joined and include a 2 bp spacer (*ii*; [24]) or when 47 nt and 65 nt strands from a 50 bp substrate and an 87 bp substrate respectively are joined with a similar spacer(*iii*). **(B) **Fractionation of purified DNA fragments from three protein-DNA complexes, tested in-gel, for cleavage activity in the presence and absence of Mg^++ ^(see Methods). Although some fragments show partial degradation, the presence of the expected HMW fragments only in the two predicted complexes and only in the presence of Mg^++^, confirms both the formation of PECs and the activity of the fusion protein. Use of the GATC sequencing reactions ladder individually and pooled (L) provided only an approximation of fragment size. Mg^++ ^provided in lane 1 as MgAc; in lane 2 as MgCl_2_. Click here for file

Additional file 2 **Composite of annotated gels showing footprinting reactions of the ends of IS2**. Cleavage patterns of: (I) footprinted (FO) top and bottom strands (IRRA and IRRB) of the right end of IS2, and (II) top and bottom strands (IRLA and IRLB) of the left end of IS2, run on 8% polyacrylamide sequencing gels, side by side with the cleaved unbound (free) DNA control reactions (FR) and the G+A Maxam-Gilbert sequencing reactions. Annotated G+A reactions identify purines with upper case letters and missing or partially visible pyrimidines with lower case letters. For the footprinted lanes, residues are identified as weakly (gray bars) or strongly (black bars) protected, using the protocol described in Figure 4. The sequences of the two strands of each end are shown beneath each corresponding pair of gels with protected residues as described above. Bands in the gels and the sequences are numbered from the outside ends to the inside ends, 1-41 for IRR and 1-42 for IRL. Square brackets identify the sequences of the ends. Negative numbers identify residues of host DNA which flank the outer ends of the termini and numbers greater than 41 in IRR and greater than 42 in IRL identify residues of IS*2 *adjacent to the inside ends of the termini. For the IRLB gel II, (*ii*) the zone of compression which masks the footprinting pattern from G5 to A-9 is shown more clearly in the inset. Click here for file

Additional file 3 **Curvature analysis of IS*2 *target sites**. Additional Predicted Curvature (PC) profiles of 200 bp fragments encompassing insertion sites (filled circles), computed by the bend.it algorithm are shown. Click here for file

Additional file 4 **Three dimensional representations of IS*2 *target regions**. These profiles correspond to 200 bp fragments flanking the insertion site (highlighted in green). The representations adopted S-like or L-like shapes. Click here for file

## References

[B1] Chandler M Mahillon J Craig NL, Craigie R, Gellert M, Lambowitz AM Insertion sequences revisited Mobile DNA II 2002 Washington, DC: ASM Press 305 366

[B2] Rousseau P Normand C Loot C Turlan C Alazard R Duval-Valentin G Chandler M Craig NL, Craigie R, Gellert M, Lambowitz AM Transposition of IS911 Mobile DNA I 2002 Washington, DC: ASM Press 367 383

[B3] Duval-Valentin G Marty-Cointin B Chandler M Requirement of IS911 replication before integration defines a new bacterial transposition pathway Embo J 2004 23 3897 3906 10.1038/sj.emboj.7600395 15359283 PMC522794

[B4] Craig NL Unity in transposition reactions Science 1995 270 253 254 10.1126/science.270.5234.253 7569973

[B5] Haren L Ton-Hoang B Chandler M Integrating DNA: transposases and retroviral integrases Annu Rev Microbiol 1999 53 245 281 10.1146/annurev.micro.53.1.245 10547692

[B6] Turlan C Chandler M Playing second fiddle: second-strand processing and liberation of transposable elements from donor DNA Trends Microbiol 2000 8 268 274 10.1016/S0966-842X(00)01757-1 10838584

[B7] Polard P Chandler M An *in vivo *transposase-catalyzed single-stranded DNA circularization reaction Genes Dev 1995 9 2846 2858 10.1101/gad.9.22.2846 7590258

[B8] Polard P Ton-Hoang B Haren L Betermier M Walczak R Chandler M IS911-mediated transpositional recombination *in vitro* J Mol Biol 1996 264 68 81 10.1006/jmbi.1996.0624 8950268

[B9] Ton-Hoang B Betermier M Polard P Chandler M Assembly of a strong promoter following IS911 circularization and the role of circles in transposition Embo J 1997 16 3357 3371 10.1093/emboj/16.11.3357 9214651 PMC1169952

[B10] Lewis LA Cylin E Lee HK Saby R Wong W Grindley ND The left end of IS2: a compromise between transpositional activity and an essential promoter function that regulates the transposition pathway J Bacteriol 2004 186 858 865 10.1128/JB.186.3.858-865.2004 14729714 PMC321474

[B11] Lewis LA Grindley ND Two abundant intramolecular transposition products, resulting from reactions initiated at a single end, suggest that IS2 transposes by an unconventional pathway Mol Microbiol 1997 25 517 529 10.1046/j.1365-2958.1997.4871848.x 9302014

[B12] Sekine Y Aihara K Ohtsubo E Linearization and transposition of circular molecules of insertion sequence IS3 J Mol Biol 1999 294 21 34 10.1006/jmbi.1999.3181 10556026

[B13] Sekine Y Eisaki N Ohtsubo E Translational control in production of transposase and in transposition of insertion sequence IS3 J Mol Biol 1994 235 1406 1420 10.1006/jmbi.1994.1097 8107082

[B14] Haas M Rak B Escherichia coli insertion sequence IS150: transposition via circular and linear intermediates J Bacteriol 2002 184 5833 5841 10.1128/JB.184.21.5833-5841.2002 12374815 PMC135391

[B15] Kiss J Olasz F Formation and transposition of the covalently closed IS30 circle: the relation between tandem dimers and monomeric circles Mol Microbiol 1999 34 37 52 10.1046/j.1365-2958.1999.01567.x 10540284

[B16] Szabo M Kiss J Nagy Z Chandler M Olasz F Sub-terminal sequences modulating IS30 transposition *in vivo *and *in vitro* J Mol Biol 2008 375 337 352 10.1016/j.jmb.2007.10.043 18022196

[B17] Berger B Haas D Transposase and cointegrase: specialized transposition proteins of the bacterial insertion sequence IS21 and related elements Cell Mol Life Sci 2001 58 403 419 10.1007/PL00000866 11315188 PMC11337337

[B18] Prudhomme M Turlan C Claverys JP Chandler M Diversity of Tn4001 transposition products: the flanking IS256 elements can form tandem dimers and IS circles J Bacteriol 2002 184 433 443 10.1128/JB.184.2.433-443.2002 11751820 PMC139565

[B19] Ton-Hoang B Polard P Haren L Turlan C Chandler M IS911 transposon circles give rise to linear forms that can undergo integration *in vitro* Mol Microbiol 1999 32 617 627 10.1046/j.1365-2958.1999.01379.x 10320583

[B20] Hu ST Hwang JH Lee LC Lee CH Li PL Hsieh YC Functional analysis of the 14 kDa protein of insertion sequence 2 J Mol Biol 1994 236 503 513 10.1006/jmbi.1994.1161 8107136

[B21] Polard P Prere MF Chandler M Fayet O Programmed translational frameshifting and initiation at an AUU codon in gene expression of bacterial insertion sequence IS911 J Mol Biol 1991 222 465 477 10.1016/0022-2836(91)90490-W 1660923

[B22] Vogele K Schwartz E Welz C Schiltz E Rak B High-level ribosomal frameshifting directs the synthesis of IS150 gene products Nucleic Acids Res 1991 19 4377 4385 10.1093/nar/19.16.4377 1653413 PMC328623

[B23] Hu ST Lee LC Lei GS Detection of an IS2-encoded 46-kilodalton protein capable of binding terminal repeats of IS2 J Bacteriol 1996 178 5652 5659 8824609 10.1128/jb.178.19.5652-5659.1996 PMC178403

[B24] Lewis LA Gadura N Greene M Saby R Grindley ND The basis of asymmetry in IS2 transposition Mol Microbiol 2001 42 887 901 10.1046/j.1365-2958.2001.02662.x 11737634

[B25] Szeverenyi I Bodoky T Olasz F Isolation, characterization and transposition of an (IS2)2 intermediate Mol Gen Genet 1996 251 281 289 8676870 10.1007/BF02172518

[B26] Normand C Duval-Valentin G Haren L Chandler M The terminal inverted repeats of IS911: requirements for synaptic complex assembly and activity J Mol Biol 2001 308 853 871 10.1006/jmbi.2001.4641 11352577

[B27] Rousseau P Tardin C Tolou N Salome L Chandler M A model for the molecular organisation of the IS911 transpososome Mob DNA 2010 1 16 10.1186/1759-8753-1-16 20553579 PMC2909936

[B28] Rousseau P Loot C Guynet C Ah-Seng Y Ton-Hoang B Chandler M Control of IS911 target selection: how OrfA may ensure IS dispersion Mol Microbiol 2007 63 1701 1709 10.1111/j.1365-2958.2007.05615.x 17367389

[B29] Reimmann C Moore R Little S Savioz A Willetts NS Haas D Genetic structure, function and regulation of the transposable element IS21 Mol Gen Genet 1989 215 416 424 10.1007/BF00427038 2540414

[B30] Kiss J Nagy Z Toth G Kiss GB Jakab J Chandler M Olasz F Transposition and target specificity of the typical IS30 family element IS1655 from *Neisseria meningitidis* Mol Microbiol 2007 63 1731 1747 10.1111/j.1365-2958.2007.05621.x 17367392

[B31] Lewis LA Astatke M Umekubo PT Alvi S Saby R Afrose J Soluble expression, purification and characterization of the full length IS2 transposase Mob DNA 2011 2 14 10.1186/1759-8753-2-14 22032517 PMC3219604

[B32] Haren L Normand C Polard P Alazard R Chandler M IS911 transposition is regulated by protein-protein interactions via a leucine zipper motif J Mol Biol 2000 296 757 768 10.1006/jmbi.1999.3485 10677279

[B33] Nagy Z Szabo M Chandler M Olasz F Analysis of the N-terminal DNA binding domain of the IS30 transposase Mol Microbiol 2004 54 478 488 10.1111/j.1365-2958.2004.04279.x 15469518

[B34] Szabo M Kiss J Olasz F Functional organization of the inverted repeats of IS30 J Bacteriol 2010 192 3414 3423 10.1128/JB.01382-09 20418401 PMC2897661

[B35] Hennig S Ziebuhr W Characterization of the transposase encoded by IS256, the prototype of a major family of bacterial insertion sequence elements J Bacteriol 2010 192 4153 4163 10.1128/JB.00226-10 20543074 PMC2916423

[B36] Bhasin A Goryshin IY Steiniger-White M York D Reznikoff WS Characterization of a Tn5 pre-cleavage synaptic complex J Mol Biol 2000 302 49 63 10.1006/jmbi.2000.4048 10964560

[B37] Stalder R Caspers P Olasz F Arber W The N-terminal domain of the insertion sequence 30 transposase interacts specifically with the terminal inverted repeats of the element J Biol Chem 1990 265 3757 3762 2154486

[B38] Hallet B Rezsohazy R Mahillon J Delcour J IS231A insertion specificity: consensus sequence and DNA bending at the target site Mol Microbiol 1994 14 131 139 10.1111/j.1365-2958.1994.tb01273.x 7830551

[B39] Vinogradov AE DNA helix: the importance of being GC-rich Nucleic Acids Res 2003 31 1838 1844 10.1093/nar/gkg296 12654999 PMC152811

[B40] Lei GS Hu ST Functional domains of the InsA protein of IS2 J Bacteriol 1997 179 6238 6243 9335268 10.1128/jb.179.20.6238-6243.1997 PMC179535

[B41] Haren L Polard P Ton-Hoang B Chandler M Multiple oligomerisation domains in the IS911 transposase: a leucine zipper motif is essential for activity J Mol Biol 1998 283 29 41 10.1006/jmbi.1998.2053 9761671

[B42] Derbyshire KM Hwang L Grindley ND Genetic analysis of the interaction of the insertion sequence IS903 transposase with its terminal inverted repeats Proc Natl Acad Sci USA 1987 84 8049 8053 10.1073/pnas.84.22.8049 2825175 PMC299474

[B43] Johnson RC Reznikoff WS DNA sequences at the ends of transposon Tn5 required for transposition Nature 1983 304 280 282 10.1038/304280a0 6306482

[B44] Makris JC Nordmann PL Reznikoff WS Mutational analysis of insertion sequence 50 (IS50) and transposon 5 (Tn5) ends Proc Natl Acad Sci USA 1988 85 2224 2228 10.1073/pnas.85.7.2224 2832849 PMC279962

[B45] Huisman O Errada PR Signon L Kleckner N Mutational analysis of IS10's outside end Embo J 1989 8 2101 2109 2551675 10.1002/j.1460-2075.1989.tb03619.x PMC401101

[B46] Zerbib D Prentki P Gamas P Freund E Galas DJ Chandler M Functional organization of the ends of IS1: specific binding site for an IS 1-encoded protein Mol Microbiol 1990 4 1477 1486 10.1111/j.1365-2958.1990.tb02058.x 1962838

[B47] Derbyshire KM Grindley ND Binding of the IS903 transposase to its inverted repeat *in vitro* Embo J 1992 11 3449 3455 1324175 10.1002/j.1460-2075.1992.tb05424.x PMC556880

[B48] Jilk RA York D Reznikoff WS The organization of the outside end of transposon Tn5 J Bacteriol 1996 178 1671 1679 8626296 10.1128/jb.178.6.1671-1679.1996 PMC177853

[B49] Ichikawa H Ikeda K Amemura J Ohtsubo E Two domains in the terminal inverted-repeat sequence of transposon Tn3 Gene 1990 86 11 17 10.1016/0378-1119(90)90108-4 2155858

[B50] Ichikawa H Ikeda K Wishart WL Ohtsubo E Specific binding of transposase to terminal inverted repeats of transposable element Tn3 Proc Natl Acad Sci USA 1987 84 8220 8224 10.1073/pnas.84.23.8220 2825182 PMC299513

[B51] New JH Eggleston AK Fennewald M Binding of the Tn3 transposase to the inverted repeats of Tn3 J Mol Biol 1988 201 589 599 10.1016/0022-2836(88)90640-7 2843651

[B52] Craigie R Mizuuchi M Mizuuchi K Site-specific recognition of the bacteriophage Mu ends by the Mu A protein Cell 1984 39 387 394 10.1016/0092-8674(84)90017-5 6094016

[B53] Zou AH Leung PC Harshey RM Transposase contacts with mu DNA ends J Biol Chem 1991 266 20476 20482 1657926

[B54] Lavoie BD Chan BS Allison RG Chaconas G Structural aspects of a higher order nucleoprotein complex: induction of an altered DNA structure at the Mu-host junction of the Mu type 1 transpososome Embo J 1991 10 3051 3059 1655409 10.1002/j.1460-2075.1991.tb07856.x PMC453020

[B55] Mizuuchi M Baker TA Mizuuchi K DNase protection analysis of the stable synaptic complexes involved in Mu transposition Proc Natl Acad Sci USA 1991 88 9031 9035 10.1073/pnas.88.20.9031 1656459 PMC52645

[B56] Surette MG Harkness T Chaconas G Stimulation of the Mu A protein-mediated strand cleavage reaction by the Mu B protein, and the requirement of DNA nicking for stable type 1 transpososome formation. *In vitro *transposition characteristics of mini-Mu plasmids carrying terminal base pair mutations J Biol Chem 1991 266 3118 3124 1847140

[B57] Gueguen E Rousseau P Duval-Valentin G Chandler M The transpososome: control of transposition at the level of catalysis Trends Microbiol 2005 13 543 549 10.1016/j.tim.2005.09.002 16181782

[B58] Chalmers R Guhathakurta A Benjamin H Kleckner N IHF modulation of Tn10 transposition: sensory transduction of supercoiling status via a proposed protein/DNA molecular spring Cell 1998 93 897 908 10.1016/S0092-8674(00)81449-X 9630232

[B59] Crellin P Chalmers R Protein-DNA contacts and conformational changes in the Tn10 transpososome during assembly and activation for cleavage Embo J 2001 20 3882 3891 10.1093/emboj/20.14.3882 11447129 PMC125557

[B60] Yanagihara K Mizuuchi K Progressive structural transitions within Mu transpositional complexes Mol Cell 2003 11 215 224 10.1016/S1097-2765(02)00796-7 12535534

[B61] Lemberg KM Schweidenback CT Baker TA The dynamic Mu transpososome: MuB activation prevents disintegration J Mol Biol 2007 374 1158 1171 10.1016/j.jmb.2007.09.079 17988683 PMC2237893

[B62] Hagerman PJ Sequence-directed curvature of DNA Annu Rev Biochem 1990 59 755 781 10.1146/annurev.bi.59.070190.003543 2197990

[B63] Plaskon RR Wartell RM Sequence distributions associated with DNA curvature are found upstream of strong E. coli promoters Nucleic Acids Res 1987 15 785 796 10.1093/nar/15.2.785 3547329 PMC340467

[B64] Nickerson CA Achberger EC Role of curved DNA in binding of *Escherichia coli *RNA polymerase to promoters J Bacteriol 1995 177 5756 5761 7592319 10.1128/jb.177.20.5756-5761.1995 PMC177394

[B65] Lewis LA Gopaul S Marsh C The non-random pattern of insertion of IS2 into the hemB gene of *Escherichia coli* Microbiol Immunol 1994 38 461 465 7968676 10.1111/j.1348-0421.1994.tb01808.x

[B66] Sengstag C Shepherd JC Arber W The sequence of the bacteriophage P1 genome region serving as hot target for IS2 insertion EMBO J 1983 2 1777 1781 6315401 10.1002/j.1460-2075.1983.tb01657.x PMC555358

[B67] Oliveira PH Prazeres DM Monteiro GA Deletion formation mutations in plasmid expression vectors are unfavored by runaway amplification conditions and differentially selected under kanamycin stress J Biotechnol 2009 143 231 238 10.1016/j.jbiotec.2009.08.002 19665504

[B68] Whiteway J Koziarz P Veall J Sandhu N Kumar P Hoecher B Lambert IB Oxygen-insensitive nitroreductases: analysis of the roles of nfsA and nfsB in development of resistance to 5-nitrofuran derivatives in *Escherichia coli* J Bacteriol 1998 180 5529 5539 9791100 10.1128/jb.180.21.5529-5539.1998 PMC107609

[B69] Kobori S Ko Y Kato M A target site for spontaneous insertion of IS10 element in pUC19 DNA located within intrinsically bent DNA Open Microbiol J 2009 3 146 150 10.2174/1874285800903010146 19812719 PMC2758499

[B70] Dixon WJ Hayes JJ Levin JR Weidner MF Dombroski BA Tullius TD Hydroxyl radical footprinting Methods Enzymol 1991 208 380 413 1664026 10.1016/0076-6879(91)08021-9

[B71] Sambrook J Fritsch EF Maniatis T Molecular Cloning, A Laboratory Manual 1989 2 Cold Spring Harbor, NY: Cold Spring Harbor Laboratory Press

[B72] Goodsell DS Dickerson RE Bending and curvature calculations in B-DNA Nucleic Acids Res 1994 22 5497 5503 10.1093/nar/22.24.5497 7816643 PMC332108

[B73] Vlahovicek K Kajan L Pongor S DNA analysis servers: plot.it, bend.it, model.it and IS Nucleic Acids Res 2003 31 3686 3687 10.1093/nar/gkg559 12824394 PMC168966

[B74] Brukner I Sanchez R Suck D Pongor S Sequence-dependent bending propensity of DNA as revealed by DNase I: parameters for trinucleotides EMBO J 1995 14 1812 1818 7737131 10.1002/j.1460-2075.1995.tb07169.x PMC398274

