# Supplementary figures and images for: Protein-DNA interactions define the mechanistic aspects of circle formation and insertion reactions in IS2 transposition
Source: Mob DNA. 2012 Jan 26;3:1. doi: 10.1186/1759-8753-3-1 (PMC3299598; doi:10.1186/1759-8753-3-1)

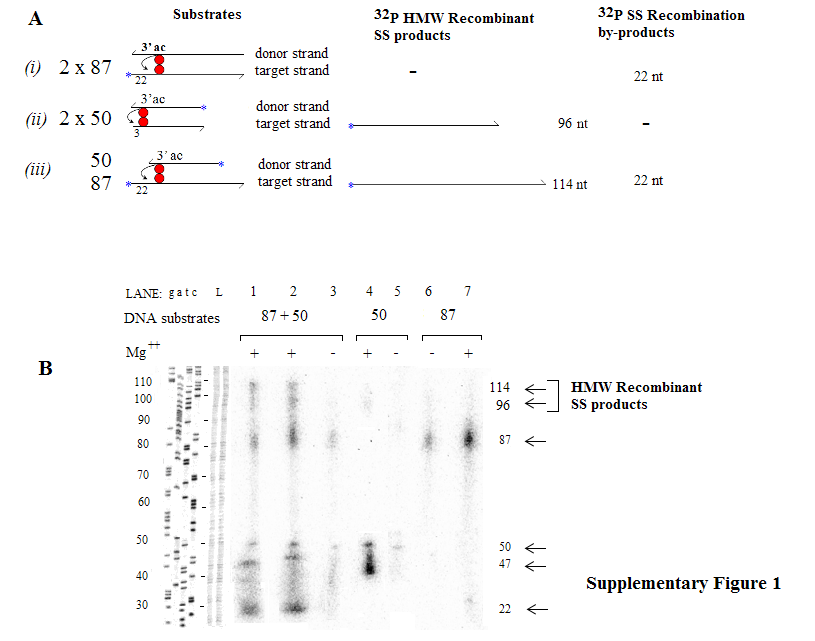

Supplement: Additional file 1 — Activity of the OrfAB-GFP fusion protein in cleavage assays with IRR substrates. (A) Schematic of expected complexes and 32P-labeled single-strand products from mixtures of double-stranded 87 bp (see description of oligonucleotides) and 50 bp [31] IRR substrates and the OrfAB-GFP protein. The 114 nt and 96 nt products would confirm the formation of paired-end complexes (PEC) and the cleavage and joining reactions of SC I (Figure 1a). For simplicity only interactions of "donor", 5' --- > CA3', and "target", 5'TG ---- > 3', strands are shown. The 87 bp substrate was labeled at the 5' end of the "target" strand and the 50 bp substrate at the 5' end of the "donor" strand. "Host DNA" sequences of 22 bp and 3 bp flanked IRR at its outside end in the 87 bp and 50 bp substrates respectively. Three possible PECs (i-iii; dimers of red spheres) and their cleavage outcomes are illustrated. The curved arrow depicts the cleaved donor strand and its transesterification attack on the target strand. Recombinant products are only predicted when two 47 nt strands from 50 bp substrates are joined and include a 2 bp spacer (ii; [24]) or when 47 nt and 65 nt strands from a 50 bp substrate and an 87 bp substrate respectively are joined with a similar spacer(iii). (B) Fractionation of purified DNA fragments from three protein-DNA complexes, tested in-gel, for cleavage activity in the presence and absence of Mg++ (see Methods). Although some fragments show partial degradation, the presence of the expected HMW fragments only in the two predicted complexes and only in the presence of Mg++, confirms both the formation of PECs and the activity of the fusion protein. Use of the GATC sequencing reactions ladder individually and pooled (L) provided only an approximation of fragment size. Mg++ provided in lane 1 as MgAc; in lane 2 as MgCl2. [file 1759-8753-3-1-S1.DOC]

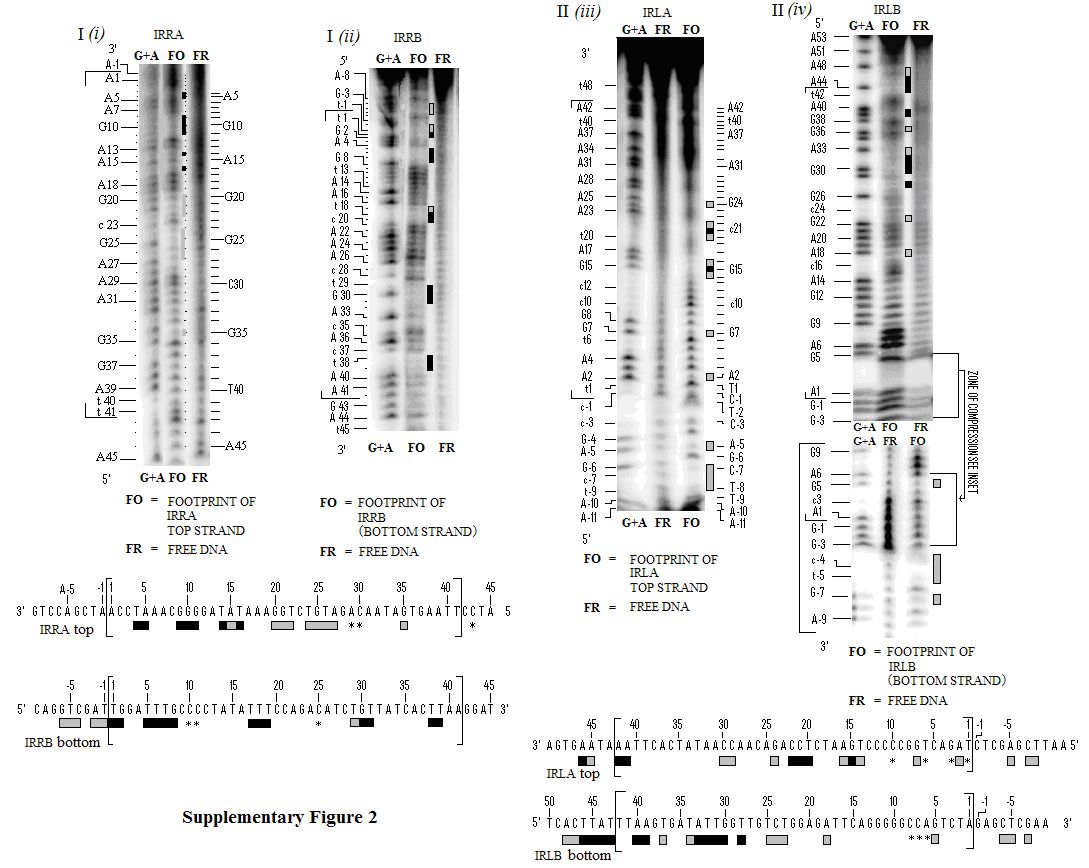

Supplement: Additional file 2 — Composite of annotated gels showing footprinting reactions of the ends of IS2. Cleavage patterns of: (I) footprinted (FO) top and bottom strands (IRRA and IRRB) of the right end of IS2, and (II) top and bottom strands (IRLA and IRLB) of the left end of IS2, run on 8% polyacrylamide sequencing gels, side by side with the cleaved unbound (free) DNA control reactions (FR) and the G+A Maxam-Gilbert sequencing reactions. Annotated G+A reactions identify purines with upper case letters and missing or partially visible pyrimidines with lower case letters. For the footprinted lanes, residues are identified as weakly (gray bars) or strongly (black bars) protected, using the protocol described in Figure 4. The sequences of the two strands of each end are shown beneath each corresponding pair of gels with protected residues as described above. Bands in the gels and the sequences are numbered from the outside ends to the inside ends, 1-41 for IRR and 1-42 for IRL. Square brackets identify the sequences of the ends. Negative numbers identify residues of host DNA which flank the outer ends of the termini and numbers greater than 41 in IRR and greater than 42 in IRL identify residues of IS2 adjacent to the inside ends of the termini. For the IRLB gel II, (ii) the zone of compression which masks the footprinting pattern from G5 to A-9 is shown more clearly in the inset. [file 1759-8753-3-1-S2.DOC]

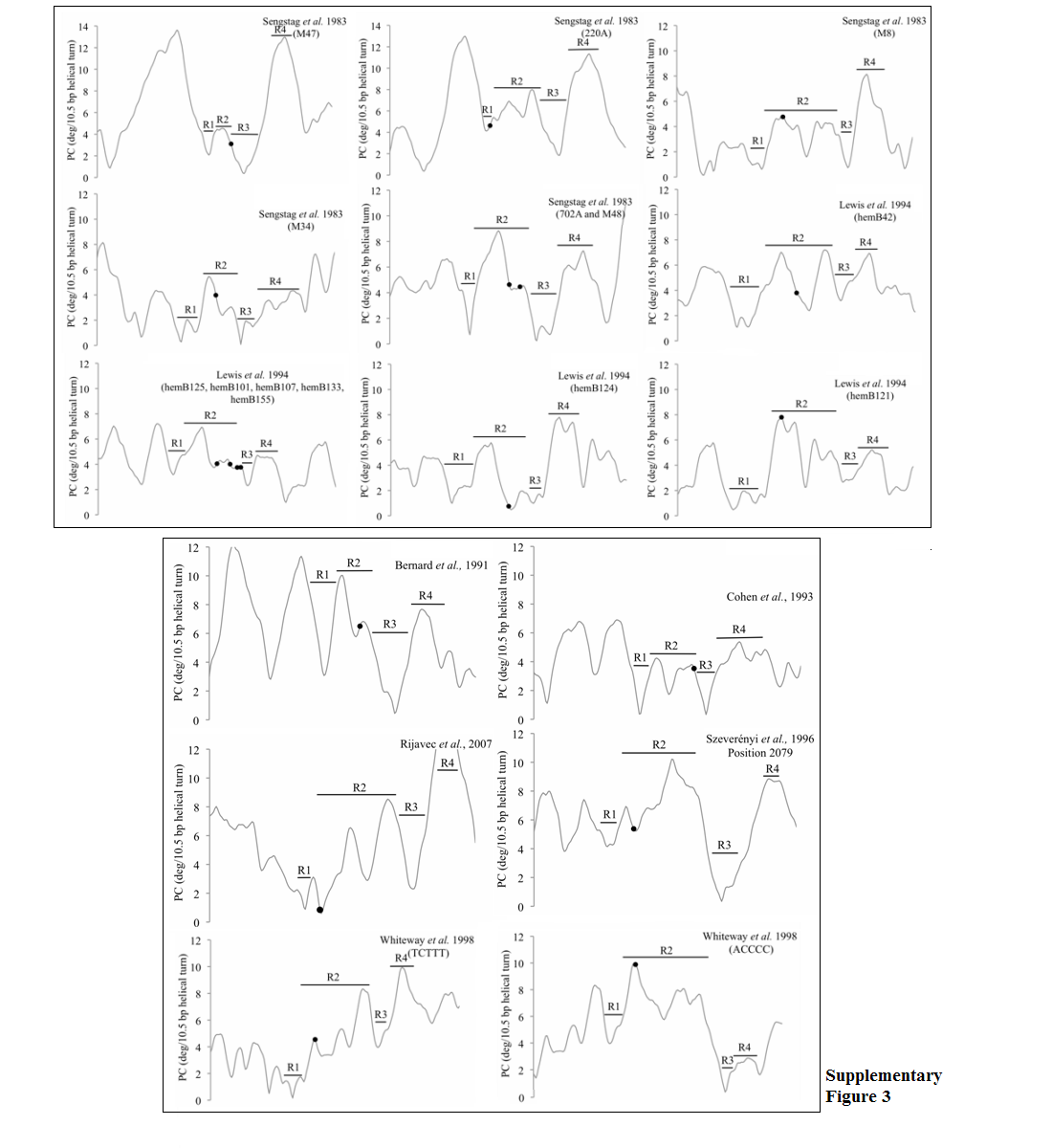

Supplement: Additional file 3 — Curvature analysis of IS2 target sites. Additional Predicted Curvature (PC) profiles of 200 bp fragments encompassing insertion sites (filled circles), computed by the bend.it algorithm are shown. [file 1759-8753-3-1-S3.DOC]

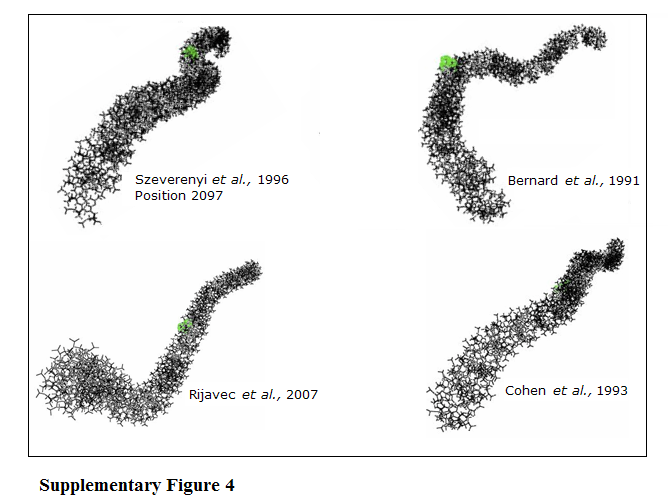

Supplement: Additional file 4 — Three dimensional representations of IS2 target regions. These profiles correspond to 200 bp fragments flanking the insertion site (highlighted in green). The representations adopted S-like or L-like shapes. [file 1759-8753-3-1-S4.DOC]
